# Pragmatic estimates of the proportion of pediatric inpatients exposed to specific medications in the USA

**DOI:** 10.1002/pds.3456

**Published:** 2013-05-23

**Authors:** Chris Feudtner, Dingwei Dai, Jennifer Faerber, Talene A Metjian, Xianqun Luan

**Affiliations:** 1 Center for Pediatric Clinical Effectiveness, The Children’s Hospital of PhiladelphiaPhiladelphia, PA, USA; 2 Antimicrobial Stewardship Program, The Children’s Hospital of PhiladelphiaPhiladelphia, PA, USA; 3 Department of Pediatrics, University of Pennsylvania School of MedicinePhiladelphia, PA, USA; 4 The Center for Clinical Epidemiology and Biostatistics, University of Pennsylvania School of MedicinePhiladelphia, PA, USA

**Keywords:** drug utilization, United States/epidemiology, pediatrics, inpatients, hospitalization, pharmacoepidemiology

## Abstract

**Purpose** To provide pragmatic national estimates of the proportion of hospitalized pediatric patients exposed to specific drugs in the USA.

**Methods** We used Premier Perspective Database and the Pediatric Health Information System data including specific drug exposures of 1.15 million inpatients <18 years old in 411 general and 52 children’s hospitals throughout the USA in 2006, extrapolating this information into the probability-based Kids’ Inpatient Database, which has demographic and clinical characteristics but no drug exposure data. We used a multivariable stratified resampling (MSR) technique to estimate the proportion of drug exposure for the 700 most commonly used drugs and performed additional stability and sensitivity analyses for 19 drugs.

**Results** The estimated proportion of pediatric inpatients exposed to specific drugs in 2006 ranged from high levels such as that of acetaminophen (17.36; 95%CI: 17.32, 17.41) to rare exposures such as bosentan (0.0018; 95%CI: 0.0013, 0.0023). Additional analyses for 19 drugs revealed that the MSR estimates were close to estimates generated by multivariable multiple imputation, with a maximum absolute difference of 0.03 for acetaminophen (17.36 vs. 17.33) and famotidine (1.90 vs. 1.93), and that even with 50% of the hospitals removed at random, the proportion estimates did not vary by more than 2.5-fold at the upper 97.5 percentile.

**Conclusions** These pragmatic national estimates of the proportion of pediatric inpatient drug exposures, generated using an MSR technique, provide a context for interpretation of drug-related adverse event reports and prioritization of pediatric pharmacology research. © 2013 The Authors. Pharmacoepidemiology and Drug Safety published by John Wiley & Sons, Ltd.

## BACKGROUND

Each year in the USA, approximately 6.39 million pediatric hospitalizations last on average 3.8 days and accumulate $105 billion of aggregate charges.^1^ During these hospitalizations, infants, children, and adolescents with a wide variety of medical conditions are treated with a variety of drugs, for both on-label and off-label indications^2^–^5^, but the national proportion of patients exposed to different drugs remains unknown.^6^ Lack of reasonable national estimates of the proportion of pediatric inpatients exposed to specific drugs has hampered the ability to monitor trends in hospital drug usage patterns and evaluate reports of adverse drug events or errors associated with specific drugs.^7^–^10^

One strategy to develop useful estimates of the proportion exposed to various drugs is to extrapolate information from one data source into the framework of another. Data regarding pediatric inpatient drug exposure exist for extensive samples of patients in children’s hospitals (the Pediatric Health Information System [PHIS]) and general hospitals (the Premier Perspective Database [PPD], which also includes several children’s hospitals), but these samples are neither random nor constructed to be nationally representative.^11^ In contrast, the Agency for Healthcare Research and Quality (AHRQ)’s Kid’s Inpatient Database (KID) is constructed, stratified, and weighted to enable the generation of probability-based estimates for the USA, but lacks inpatient drug exposure data.^12^ All these data sources have patient demographic and clinical information, such as age, length of stay (LOS), diagnosis (recorded as an All Patient Refined Diagnostic Related Group [APR-DRG]), and hospital type (children’s or general hospitals), characteristics that we have found are associated with the likelihood of inpatient drug exposures.^13^ One can then conceptualize the extrapolation in an analytic framework where each of the three data sources is incomplete, but together they are complementary (Supplemental Figure A).

In conceptualizing the KID as missing data regarding drug exposure, the mechanism underlying this missing data is known: all subjects in KID are missing drug exposure data. What is not completely understood is how patients and patterns of care recorded in KID differ from those in the PHIS and Premier, both in terms of individual patient demographic and clinical characteristics and in terms of the hospitals (and consequently, physicians, healthcare teams, and practice patterns) sampled in these different sample frames, how these differences affect the likelihood that specific patients will be exposed to specific drugs, and whether these individual differences affect the population-level average proportion exposed estimates.

In this study, we used a multivariable stratified resampling (MSR) procedure,^14^,^15^ based on the four strata of age, LOS, hospital type, and APR-DRG, to generate national-level estimates of the proportion of pediatric inpatient exposure for 700 of the most commonly used drugs, and then for 19 selected drugs, we assessed the stability of these estimates compared with a multivariable multiple imputation (MMI) procedure^16^,^17^ and performed two sensitivity analyses to quantify the potential range of estimation error due to patient sampling error and hospital composition error.

## METHODS

The Children’s Hospital of Philadelphia’s Institutional Review Board approved this study.

### Data sources

We used three primary data sources. First, PHIS (Children’s Hospital Association, Kansas City, KS) comprises administrative discharge data from children’s hospitals for major metropolitan areas across the USA. Second, PPD (Premier, Inc, San Diego, CA) comprises data from a broad array of academic medical centers, community-based hospitals, and large multi-hospital systems. For this study, 40 hospitals in PHIS and 423 hospitals in the PPD in 2006 contained detailed pharmacy information for each day of the hospital stay.^11^ Third, KID is a probability-based sample of inpatient pediatric admissions from all hospitals that provide data to AHRQ. KID employs a complex sampling scheme, enabling generation of national estimates in the 2006 KID; 38 states participated, with 80% of pediatric hospitalizations randomly chosen from each participating hospital, except for “normal newborns,” of whom 20% were randomly selected from each hospital. Of the KID hospitalizations, 7% are from children’s and 93% from general hospitals. The KID only identifies discrete hospitalizations, not unique patients, and does not contain pharmacy information.^12^ Together, PHIS and Premier constitute 19.9% of all pediatric hospitalizations for 2006.

### Data management

We categorized PHIS and PPD records into children’s hospitals and general hospitals. From PPD, records from two hospitals included in PHIS were omitted; 12 hospitals identified as children’s hospitals and exhibiting demographics consistent with those observed in children’s hospitals in the PHIS and KID databases were classified as children’s hospitals; the remaining hospitals were classified as general hospitals. We implemented a standardized dictionary of generic drug entities, specified by 1227 distinct codes in PHIS and 1564 in PPD. After harmonizing terminology, PHIS had 1144 distinct codes and PPD 1337.^13^

### Comparison of PHIS and Premier samples to KID sample

We first described the demographic and clinical characteristics of the sample by calculating percentages (in KID, accounting for the survey design) and computed the standardized proportion differences between PHIS/Premier and KID.^18^ We calculated the percentage of hospitalizations for each APR-DRG in all three databases and measured the difference between the children’s hospitals in PHIS/PPD and in KID, doing likewise for general hospitals. We also assessed the proportion of hospitalizations in PHIS/PPD that had multivariable matches in KID, and vice versa.

### Multivariable stratified resampling

Stratified resampling was used to generate national estimates of drug exposure for 700 drugs (Supplementary Figure B), stratifying PHIS/Premier data and KID data into 7068 strata based on patient age (<1, 1–4, 5–12, and 13–17 years), LOS (1, 2–7, and >7 days), APR-DRG (315 observed), and hospital type (general and children’s), with 74.83% of the strata present in both PHIS/Premier and KID. For each hospitalization in KID, we randomly sampled with replacement a corresponding stratified record from PHIS/Premier.^19^ Within each matched pair, the KID record was updated with the drug exposure status from PHIS/Premier. We resampled 1000 times and examined the distribution of exposure for each of the 700 drugs. We generated national proportion exposed estimates by taking the average exposure (across the 1000 samples) weighted by the KID discharge weight.

### Multivariable multiple imputation

For 19 drugs selected across the range of the proportions of patients exposed (Supplementary Figure C), we performed multiple imputation as a stability analysis^20^ of the stratified resampling approach. We combined the three databases and imputed missing values for drug exposure in the KID database using PHIS and Premier records with no missing values regarding age, LOS, hospital type, APR-DRG, and drug exposure (<1% of the combined sample had missing values for any of the non-drug covariates). For each of the 19 drugs, we fit separate data augmentation models using Markov Chain Monte Carlo algorithms (which has been shown in simulations to perform adequately for imputing binary variables^17^), fitting separate models for children’s and general hospitals, and then combined the estimates. To obtain national estimates of drug exposure for the KID observations, we computed the mean probability of drug exposure using the KID sample weights and accounting for the KID survey structure to correctly estimate the variances, then compared the estimates generated by these techniques.

### Sensitivity analysis regarding proportion of patients cared for at children’s hospitals

Because children treated in children’s and general hospitals differ in terms of demographic, clinical, and drug usage patterns, extrapolation methods must account for these differences to reflect the national case mix.^13^ To assess the consequences of a source population that deviates from the national case mix, we performed sensitivity analyses for each of the 19 drugs (Supplemental Figure D). We drew a random subset of 100 000 records from PHIS/Premier, such that 7% of the patients were treated at children’s hospitals and 93% at general hospitals (which is the percentage observed in KID), and considered this database a new “base case” sample. We then drew another random “modified” sample of 100 000 records from PHIS/Premier, systematically varying the percentage of patients treated at children’s hospitals from 0% to 14%. The difference in the proportion of observed drug exposure between the “modified” sample and the “base case” sample was noted for each of the 19 drugs. We repeated this procedure 1000 times, resampling with replacement, for each percentage of children’s hospitals. We evaluated the effect of the sample modifications by comparing the average differences between the modified and base case estimates.

### Sensitivity analysis of specific hospitals’ influence on proportion exposed estimates

Hospitals vary in their use of specific drugs, raising concern that over-sampling or under-sampling of high-utilization hospitals could distort national averages.^13^ To assess the magnitude of this potential distortion, we developed and implemented a “hospital knockout” methodology (Supplemental Figure E). Specifically, we first randomly eliminated a fixed percentage of the hospitals (10%, 25%, or 50%) in PHIS/Premier. We then matched each KID record to a record from PHIS/Premier and replaced the missing drug exposure data in the KID record with the data from PHIS/Premier. We resampled with replacement 1000 times, as detailed earlier, to generate national estimates of the proportion of inpatient drug exposure for each of the 19 drugs. For each drug, we examined how the percentage of hospitals “knocked out” from PHIS/Premier affected drug exposure estimates.

### Statistical software

All data management and analyses were conducted using sas 9.3 (SAS Institute Inc., Cary, NC) and Stata 12.1 (StataCorp, College Station TX).

## RESULTS

**Figure 1:**
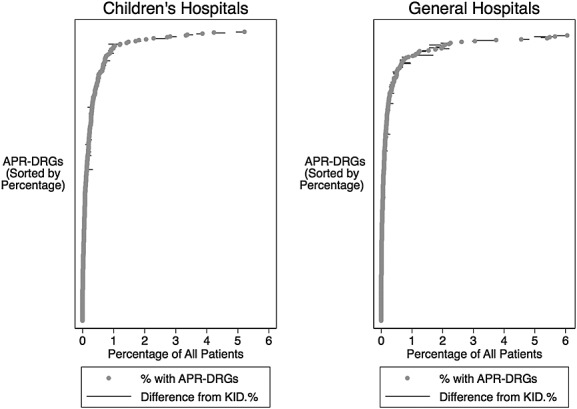
Comparison of proportion of patients in 315 APR-DRGs

Compared with the KID children’s and general hospitals, characteristics of patients in our sample were qualitatively similar in terms of demographic characteristics such as age, gender, and race, hospital location and teaching status, and insurance payer, and clinical characteristics such as LOS and disposition (Table 1). In terms of medical conditions, the median difference in the proportion of patients in each of 315 APR-DRG groups in the combined databases and the KID was 0.01 (maximum, 0.48) in children’s hospitals and 0.01 (maximum, 0.69) in general hospitals (Figure 1). In terms of the degree to which the samples had similar subjects (defined by the four stratification variables of age, LOS, APR-DRG, and hospital type), 99.2% of the hospitalized subjects in KID were able to be matched to patients in PHIS/Premier, and over 99.9% of patients in PHIS/Premier were matched with KID patients.

**Table 1:** Characteristic of subjects in children’s and general hospitals compared with Kid’s Inpatient Database (KID)

	Children’s hospitals			General hospitals		
	Study sample (hospitals, *n* = 52)	KID* (hospitals, *n* = 36)	Standardized difference†	Study sample (hospitals, *n* = 411)	KID* (hospitals, *n* = 3703)	Standardized difference†
Characteristics	(Hospitalizations, *n* = 530 708)	(Hospitalizations, *n* = 476 508)		(Hospitalizations, *n* = 782 344)	(Hospitalizations, *n* = 6 138 431)	
Age (years)			0.0166			0.2602
<1	34.8%	32.3%		80.0%	75.5%	
1–4	23.3%	24.7%		6.2%	7.5%	
5–9	15.6%	16.8%		3.3%	4.4%	
10–14	15.7%	16.1%		3.7%	4.9%	
15–17	10.6%	9.9%		6.8%	7.7%	
Gender			0.0603			0.0040
Male	54.7%	55.0%		50.6%	50.8%	
Female	45.3%	45.0%		49.4%	49.2%	
Race‡			0.0770			0.0448
White	47.6%	43.8%		49.5%	51.7%	
Hispanic	16.9%	31.1%		11.6%	23.9%	
Black	20.8%	14.8%		15.0%	14.5%	
Asian/Pacific Islander	2.3%	2.9%		2.7%	3.7%	
American Indian	0.4%	0.5%		0.9%	0.7%	
Other	12.0%	6.8%		20.4%	5.5%	
Region			0.1641			0.2196
Midwest	28.1%	27.4%		18.4%	21.3%	
Northeast	16.0%	4.8%		12.0%	18.0%	
South	37.6%	29.5%		49.5%	38.7%	
West	18.3%	38.3%		20.1%	22.1%	
Urban–rural			0			0.0324
Urban	100%	100.0%		88.9%	87.9%	
Rural	0%	0%		11.1%	12.1%	
Teaching status			0.4205			0.2741
Teaching	97.7%	90.1%		37.6%	51.3%	
Non-teaching	2.3%	9.9%		62.4%	48.7%	
Length of stay			0.0016			0.0964
Days^1^	3 (2–5)	3 (2–5)		2 (2–3)	2 (2–4)	
1 day	23.12	23.27		18.79	20.65	
2–7 days	61.02	60.39		75.75	73.27	
>7 days	15.87	16.34		5.46	6.08	
Payers			0.0600			0.0704
Medicaid	43.8%	46.8%		40.5%	44.0%	
Other government payers	3.6%	0.2%		1.7%	0.2%	
Non-government insurance	49.7%	44.9%		50.8%	47.8%	
Self-pay	2.8%	3.4%		4.6%	5.0%	
No charge	0.1%	0.1%		0.2%	0.2%	
Other	0.0%	4.6%		2.2%	2.9%	
Disposition			0.0389			0.0049
Home	94.1%	93.4%		94.0%	93.9%	
Short-term hospital	1.1%	1.3%		1.5%	1.6%	
Home health care	2.4%	3.3%		3.6%	3.2%	
Against medical advice	0.1%	0.1%		0.1%	0.1%	
Died in hospital	1.0%	0.9%		0.3%	0.3%	
Other	1.0%	1.0%		0.5%	0.8%	

* All measures were weighted by discharged weight.

† Standardized difference calculated as the difference in proportions divided by the standard error.

‡ Race values missing in 22% of KID children’s hospitals and 27% of KID general hospitals.

1 Data expressed as median (interquartile range).

### National estimates of drug exposure

We used the MSR procedure, following the steps described in the Methods section, to generate national estimates for 700 drugs with the highest levels of exposure using the combined PHIS and Premier datasets, but corresponding to the KID database case mix. Estimates of exposure for 25 leading drugs are reported in Table 2 and for all 700 drugs (Supplemental Table A).

**Table 2:** Estimated proportion of patients exposed for 25 leading medications*

Drug	Estimated proportion exposed	95%CI	
Acetaminophen	17.36	17.32	17.41
Lidocaine	10.91	10.85	10.96
Ampicillin	9.00	8.96	9.03
Morphine	7.87	7.85	7.89
Fentanyl	7.86	7.85	7.88
Ceftriaxone	7.28	7.26	7.30
Ibuprofen	7.02	7.00	7.05
Gentamicin	6.62	6.59	6.65
Albuterol	6.47	6.45	6.48
Potassium chloride	6.01	5.99	6.03
Midazolam	5.96	5.95	5.98
Ondansetron	5.92	5.90	5.93
Propofol	5.27	5.25	5.28
Heparin	4.99	4.97	5.01
Cefazolin	4.24	4.23	4.26
Methylprednisolone	3.87	3.85	3.88
Ranitidine	3.86	3.85	3.88
Diphenhydramine	3.55	3.53	3.56
Lidocaine and prilocaine	3.41	3.38	3.44
Dexamethasone	3.09	3.08	3.11
Promethazine	2.82	2.81	2.84
Metoclopramide	2.75	2.74	2.77
Ketorolac	2.71	2.69	2.72
Cefotaxime	2.70	2.68	2.71
Calcium gluconate	2.69	2.68	2.71

* Medications used commonly for newborn infant care, specifically vitamin K, erythromycin, hepatitis B vaccine, and triple dye, are not included.

### Stability analysis for alternative estimation procedures

To perform a stability analysis and compare the estimates generated by the MSR technique to an alternative procedure, we selected 19 drugs for further evaluation, ranging from high to low levels of exposure, and from either equivalent levels of exposure in children’s and general hospitals or higher levels in either children’s or general hospitals (Table 3, columns labeled “Study sample”). We then performed MMI to generate the estimated weighted national proportions of exposure to each of the 19 drugs, reflecting the patient and hospital case mix observed in the KID database (Table 3, column labeled “Multiple imputation”). We then compared the estimates of the MSR (Table 3, column labeled “Stratified resampling”) and the MMI procedures both in absolute and relative terms (Table 3, columns at right labeled “Absolute difference” and “Ratio”) for the 19 drugs. The absolute differences in the proportion of patients exposed (MRS − MMI) across the 19 drugs ranged from −0.03 to 0.03 and, in general, decreased in magnitude as the estimated proportion of drug exposure decreased. The relative difference of the results of the two estimation techniques (stratified resampling estimate divided by multiple imputation estimate) ranged from 0.87 to 1.14.

**Table 3:** Proportion of drug exposure extrapolations for 19 drugs comparing stratified resampling and multiple imputation

	Study sample		Extrapolated national estimates		Comparison of estimates	
	Percentage of patients exposed		Multivariable stratified resampling (MSR)	Multivariable multiple imputation (MMI)	Absolute difference	Ratio
Drug	General	Children’s	% Exposed (95%CI)	% Exposed (95%CI)	MRS − MMI	MRS/MMI
Acetaminophen	13.65 (13.59, 13.74)	37.95 (37.82, 39.09)	17.36 (17.32, 17.41)	17.33 (17.20, 17.46)	0.04	1.00
Ampicillin	8.62 (8.56, 8.69)	13.94 (13.84, 14.03)	9.00 (8.96, 9.03)	8.98 (8.91, 9.07)	0.02	1.00
Levalbuterol	1.95 (1.92, 1.98)	2.53 (2.49, 2.58)	2.33 (2.31, 2.34)	2.31 (2.25, 2.38)	0.02	1.01
Famotidine	1.53 (1.49, 1.55)	2.27 (2.23, 2.31)	1.90 (1.89, 1.92)	1.93 (1.88, 1.98)	−0.03	0.98
Olanzapine	0.10 (0.09, 0.11)	0.25 (0.24, 0.26)	0.179 (0.175, 0.183)	0.171 (0.156, 0.185)	0.008	1.05
Esomeprazole	0.13 (0.12, 0.14)	0.23 (0.21, 0.24)	0.176 (0.172, 0.180)	0.176 (0.166, 0.186)	0	1.00
Clarithromycin	0.09 (0.08, 0.09)	0.15 (0.14, 0.16)	0.108 (0.105, 0.111)	0.109 (0.100, 0.118)	−0.001	0.99
Ticarcillin	0.004 (0.002, 0.005)	0.79 (0.76, 0.81)	0.068 (0.066, 0.071)	0.068 (0.062, 0.073)	0	1.00
Ganciclovir	0.005 (0.004, 0.007)	0.34 (0.32, 0.35)	0.033 (0.032, 0.035)	0.034 (0.030, 0.039)	−0.001	0.97
Tetracycline	0.013 (0.011, 0.016)	0.015 (0.012, 0.019)	0.015 (0.014, 0.016)	0.016 (0.010, 0.022)	−0.001	0.94
Prazosin	0.0090 (0.0070, 0.011)	0.021 (0.017, 0.025)	0.016 (0.014, 0.017)	0.014 (0.0098, 0.018)	0.002	1.14
Dialysis Solution	0.0080 (0.0060, 0.011)	0.058 (0.051, 0.065)	0.018 (0.017, 0.019)	0.018 (0.012, 0.023)	0	1.00
Drotrecogin alpha	0.0015 (0.0006, 0.0024)	0.086 (0.078, 0.094)	0.0084 (0.0075, 0.0094)	0.0077 (0.0046, 0.0109)	0.0007	1.09
Micafungin	0.0005 (0.0001, 0.0010)	0.081 (0.073, 0.088)	0.0069 (0.0060, 0.0077)	0.0068 (0.0043, 0.0094)	0.0001	1.01
Basiliximab	0.0005 (0.0001, 0.0010)	0.037 (0.032, 0.042)	0.0052 (0.0047, 0.0058)	0.0060 (0.0045, 0.0076)	−0.0008	0.87
Epoprostenol	0.0008 (0.0002, 0.0016)	0.050 (0.045, 0.056)	0.0049 (0.0043, 0.0056)	0.0050 (0.0027, 0.0073)	−0.0001	0.98
Rosiglitazone	0.0030 (0.0010, 0.0040)	0.002 (0.001, 0.003)	0.0038 (0.0032, 0.0045)	0.0036 (0.0010, 0.0062)	0.0002	1.06
Pravastatin	0.0005 (0.0001, 0.0010)	0.034 (0.029, 0.038)	0.0029 (0.0024, 0.0035)	0.0031 (0.0010, 0.0051)	−0.0002	0.94
Estradiol	0.0020 (0.0010, 0.0030)	0.005 (0.003, 0.007)	0.0029 (0.0024, 0.0034)	0.0027 (0.00057, 0.0049)	0.0002	1.07

### Sensitivity analyses of impact of patient and hospital composition of sample

A major concern about extrapolating national estimates from a given large sample of patients and hospitals is that a subpopulation may be over-represented or under-represented, causing a distortion. We sought to assess its potential magnitude with two additional assessments using stratified resampling.

**Figure 2:**
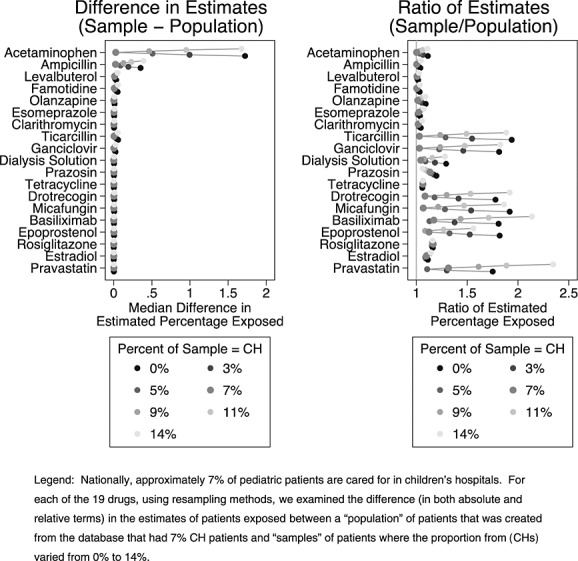
Assessment of impact on accuracy of estimated percentage of patients exposed to 19 drugs depending upon the children’s hospital composition of the sample

First, we examined the impact of having a smaller or larger proportion of patients treated in children’s hospitals in the sample, compared with the 7% proportion observed in the KID. The absolute median differences in the estimated proportion of drug exposure, across samples ranging from 0% to 14% children’s hospital patients, were again largest for the drugs with the higher levels of exposure, with differences ranging from 1.7 to 0.0002 (Figure 2, left panel). The ratios of the estimates, by contrast, were in general larger for the lower exposure drugs, ranging from 1.0 to 2.3. Some estimates of drugs with lower exposure proportions were stable across the range of children’s hospital patients (such as tetracycline, estradiol, and rosiglitazone; Figure 2, right panel).

**Figure 3:**
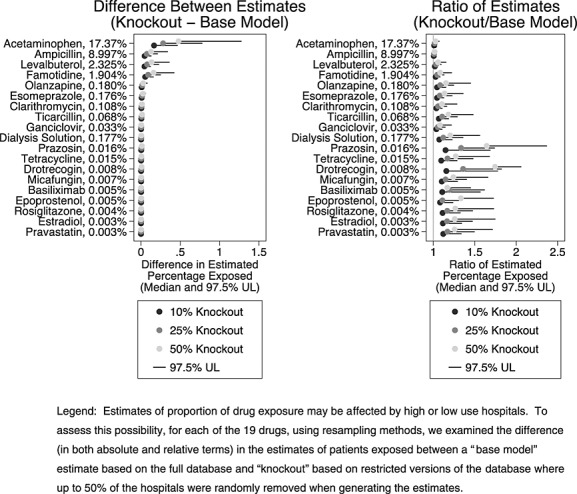
Assessment of the stability of drug exposure estimates when 10%, 25%, and 50% of hospitals were randomly knocked out of estimation sample

Second, we examined the impact of the inclusion or exclusion of specific hospitals on the stability of the estimates by employing a “hospital knockout” procedure. We observed substantial stability of the estimates with removal of 10%, 25%, and 50% of the hospitals, with absolute differences less than 0.5 and ratios of estimates of less than 1.5 (Figure 3).

## DISCUSSION

Our study, using data from the PHIS and Premier databases and extrapolating from the KID sample framework, provides national-level estimates of the proportion of pediatric inpatients exposed to 700 specific drugs that are the most common exposures. Similar to other estimates of the 10 most common drugs,^6^ acetaminophen is the drug to which the largest proportion of inpatients are exposed (17.36%), while lidocaine, ampicillin, morphine, fentanyl, ceftriaxone, ibuprofen, gentamicin, and albuterol are all on both top 10 lists, although with modestly different point estimates of the proportion of patients exposed. Descending downward from these common drugs, our estimation procedure was used for far less commonly used drugs, such as halothane, with an estimated proportion exposed of 0.0019% (95%CI: 0.0014%, 0.0024%).

Are our methods capable of providing reasonably accurate national-level estimates of these exposures in the USA? In this study, we approached this question from three directions. First, we demonstrated the similarity of the PHIS and Premier patients, regarding demographic and clinical characteristics, to those in the probability-based KID database. Second, we compared two different methods of extrapolating the drug exposure data about numerous stratified subgroups of patients from the PHIS and Premier records into the corresponding subgroups in the KID database and found that multivariable regression-based multiple imputation yielded estimates that were very close to the multivariable stratified resampling estimates. Third, we performed sensitivity analyses to determine the susceptibility of the estimates to manipulation of the source data (PHIS/Premier) in ways often cited as concerns for generating national pediatric estimates, namely changing the ratio of children’s versus general hospitals and randomly eliminating up to 50% of the source hospitals, and observed that drug exposure estimates were more concordant when the children’s hospital to general hospital ratio reflected the national ratio and were remarkably stable with random elimination of hospitals, lessening the concern that several outlier hospitals may dramatically effect national-level estimates.

These sensitivity analyses address the cardinal limitations of this study, namely that there may be differences between the hospitals that do and do not contribute to the PHIS and Premier dataset, and that such differences might bias the national estimates obtained by the procedures we have used. Although our additional analyses demonstrate the stability of the exposure estimates despite systematic alteration of the PHIS and Premier source data, without a comparison set of “gold standard” exposure measurements, the accuracy of our estimates will remain somewhat uncertain. We also examined only 19 different drugs in detail, which although selected to span the range of incidence and divergence of usage, may not behave the way that other drugs might.

In light of these limitations, how might these estimates of the proportion of pediatric inpatients exposed to specific drugs be appropriately used? For pediatric pharmacovigilance, these estimates help establish a range of the proportion of inpatients exposed to specific drugs, against which to examine the number of adverse drug events, and thus helps indicate whether to further investigate drug safety.^4^,^6^ For prioritizing pediatric drug research, the proportion of patients exposed can provide one parameter for prioritization. To be useful, the estimates must be sufficiently accurate and precise at the population level to aid decision makers, who will have to determine whether the estimates are sufficiently “reasonably accurate” for the purposes of the decisions they confront. This standard is different from what would be required to draw inferences at the individual level based on drug exposure extrapolations using either the imputation or the resampling approaches; population-level estimates, benefiting from the statistical phenomenon underlying the central limit theorem, are more robust than individual-level estimates.

Our findings also underscore two core aspects of pediatric inpatient drug usage. First, because patients cared for at children’s versus general hospitals differ substantially, in terms of ages, conditions, and patterns of drug usage^13^, and as we showed here that national-level estimates of drug exposure vary depending upon the ratio of general-to-children’s hospitals in the sample, these estimates should be generated using a nationally representative ratio. Second, as indicated by the “hospital knockout” sensitivity analysis, some drugs are used in a consistent manner across hospitals (such as ampicillin and gancylovir), whereas others display greater heterogeneity of use (such as prazosin and drotecogen alpha [which was in use in 2006 prior to being recently removed from the market]). This evidence of hospital-level variation in the use of specific drugs points to potential areas of research and quality improvement but has a minimal impact on the national-level estimates, which center around the average usage across all hospitals.

We believe that the pediatric inpatient drug exposure estimates provided by this analysis, as well as the methods used, are useful for pediatric pharmacovigilance and drug research prioritization, both for generating national-level exposure estimates and for testing the sensitivity of these estimates to potential limitations of the source data.

## CONFLICT OF INTEREST

The authors declare that they have no conflicts of interest.

**KEY POINTS**

- National-level epidemiological studies of adverse drug events require accurate estimates of drug exposure rates.
- Our study demonstrates the use of multivariable stratified resampling techniques to generate exposure estimates for 700 drugs for the United States pediatric inpatient population, and that these estimate are resistant to a variety of potential problems with the underlying data.
- These national exposure estimates can be used to inform further research into adverse drug events and drug-drug interactions among hospitalized children.
