# Supplementary material for: Pragmatic estimates of the proportion of pediatric inpatients exposed to specific medications in the USA
Source: Pharmacoepidemiol Drug Saf. 2013 May 23;22(8):890–8. doi: 10.1002/pds.3456 (PMC3810715; doi:10.1002/pds.3456)
Supplement: Supplementary file 1 [file pds0022-0890-sd1.pdf]

Pragmatic Estimates of the Proportion of Pediatric Inpatients Exposed to Specific Medication in the United States

Feudtner, Dai, Faerber, Metjian, and Luan

Supplemental Figures:

Figure A: Conceptual Model of Extrapolation Estimate Approach

Figure B: Stratified Resampling Procedure

Figure C: Log Counts of All Generic Drugs and the 19 Selected Generic Drugs

Figure D: Sensitivity Analyses of the Impact of Varying the Percentage of Patients in the Sample Treated at Children's Hospitals

Figure E: Sensitivity Analyses of the Impact of Specific Hospitals within the Sample

Supplemental Tables:

Table A: Estimated Percentage of Patients Exposed to the 700 Most Commonly Used Medications, Sorted by Percentage

Table B: Estimated Percentage of Patients Exposed to the 700 Most Commonly Used Medications, Sorted Alphabetically

Figure A - Conceptual Model of Extrapolation Estimate Approach

	Patient Demographic & Clinical Data			Sample Frame		Sampling Method	Drug Information		
**Database**	Age	LOS	APR-DRG	Children's Hospital	General Hospital	Structure	Acetamin-ophen	Ampicillin	Lev-albuterol
PHIS	✓	✓	✓	Partial	Missing	Complete	✓	✓	✓
Premier	✓	✓	✓	Minimal	Partial	Complete	✓	✓	✓
KID	✓	✓	✓	NIS	NIS	Weight PSU Strata	*Estimate 1*	*Estimate 2*	*Estimate 3*

Legend: PHIS, Pediatric Health Information System; Premier, Perspective Data Warehouse; KID, Agency for Healthcare Research and Quality's Healthcare Cost and Utilization Project's Kid's Inpatient Database; NIS, National Inpatient Sample; PSU, probability sampling unit.

Figure B – Stratified Resampling Procedure

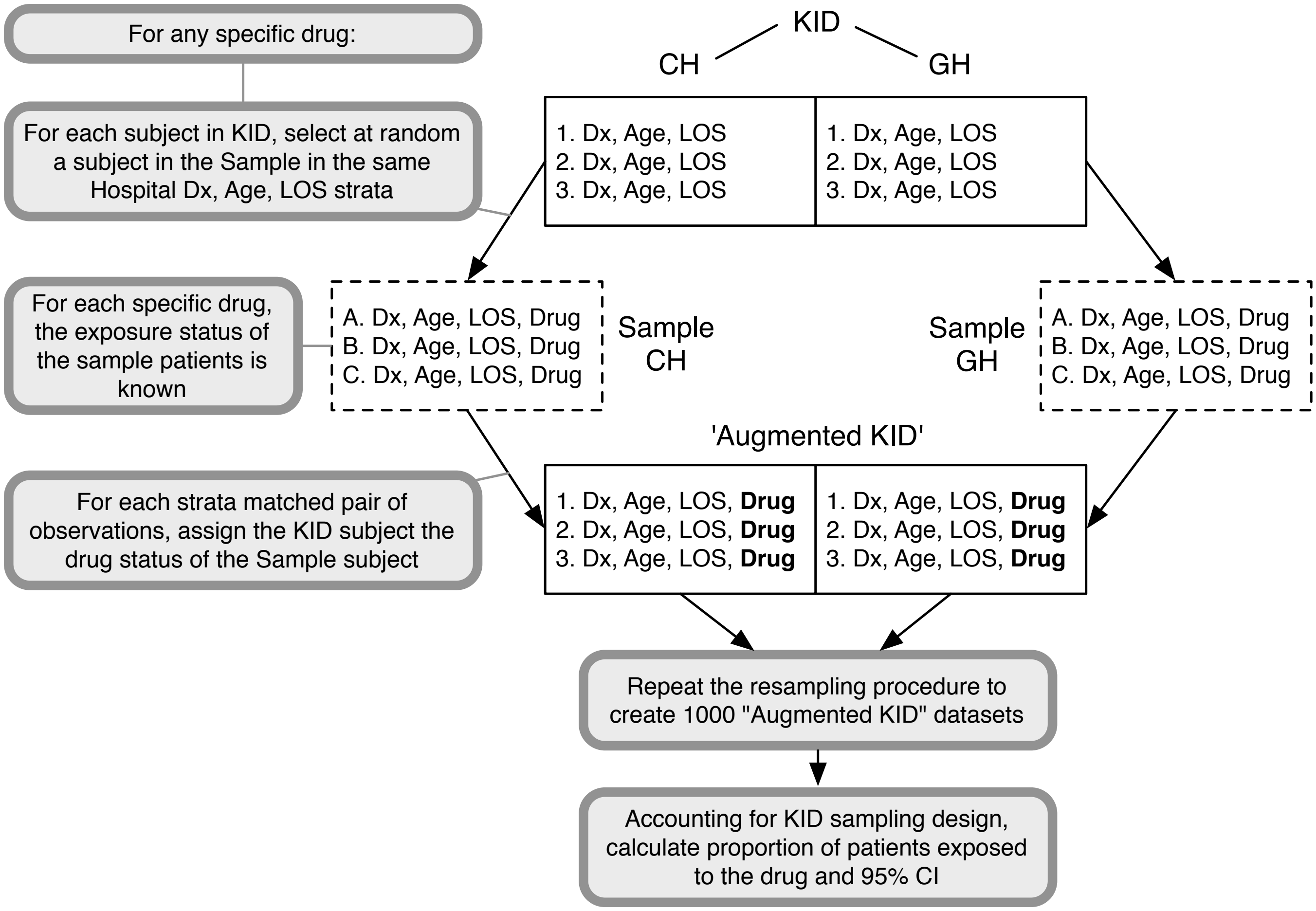

Legend: KID, Kids Inpatient Database; CH, children's hospitals; GH, general hospitals; DX, diagnosis as per the All Patient Refined Diagnostic Related Group; LOS, length of stay.

Figure C – Log Counts of All Generic Drugs and the 19 Selected Generic Drugs

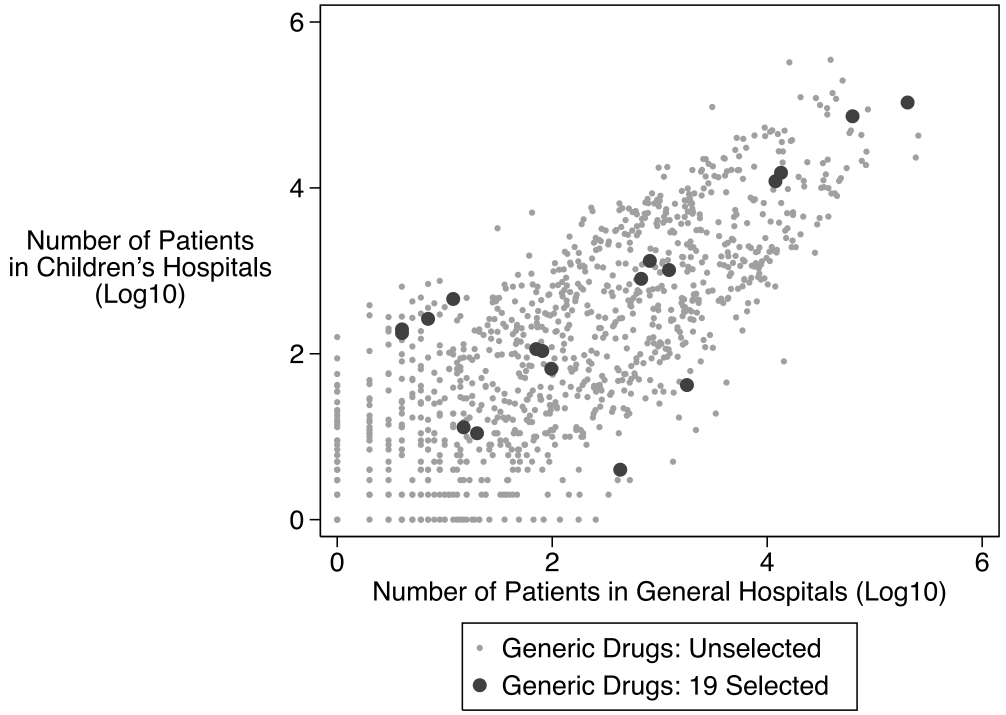

Figure D – Sensitivity Analyses of the Impact of Varying the Percentage of Patients in the Sample Treated at Children's Hospitals

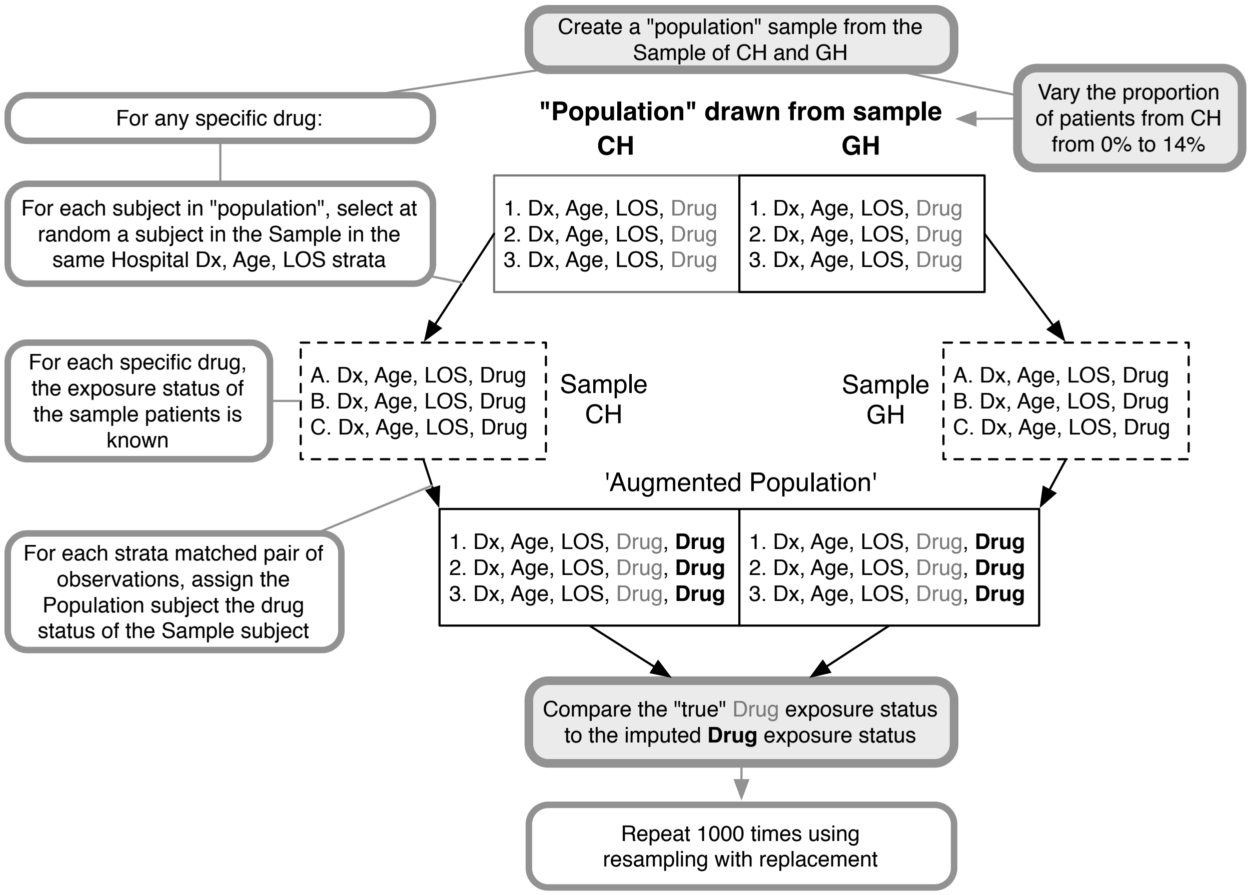

Legend: KID, Kids Inpatient Database; CH, children's hospitals; GH, general hospitals; DX, diagnosis as per the All Patient Refined Diagnostic Related Group; LOS, length of stay.

Figure E – Sensitivity Analyses of the Impact of Specific Hospitals within the Sample

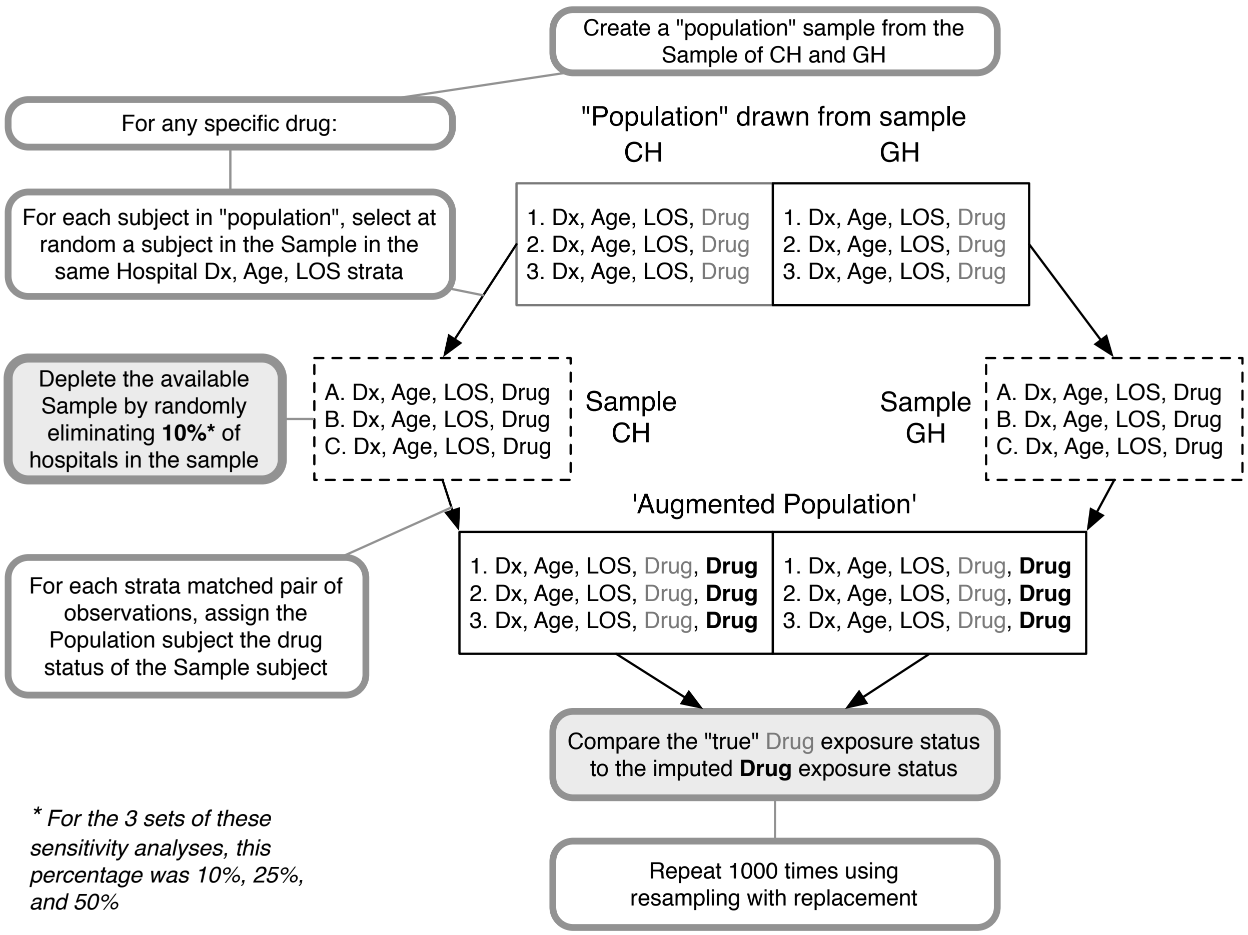

Legend: KID, Kids Inpatient Database; CH, children's hospitals; GH, general hospitals; DX, diagnosis as per the All Patient Refined Diagnostic Related Group; LOS, length of stay.

Table A: Estimated Percentage of Patients Exposed to the 700 Most Commonly Used Medications, Sorted by Percentage

Drug	Estimate	95% CI LL	95% CI UL
Acetaminophen	17.3647	17.3165	17.4138
Lidocaine	10.9071	10.8456	10.9614
Ampicillin	8.9962	8.9632	9.0285
Morphine	7.8720	7.8523	7.8894
Fentanyl	7.8643	7.8463	7.8825
Ceftriaxone	7.2806	7.2601	7.3014
Ibuprofen	7.0231	7.0014	7.0477
Gentamicin	6.6243	6.5943	6.6544
Albuterol	6.4663	6.4473	6.4833
Potassium chloride	6.0098	5.9871	6.0320
Midazolam	5.9647	5.9450	5.9831
Ondansetron	5.9153	5.8978	5.9336
Propofol	5.2665	5.2502	5.2826
Heparin	4.9935	4.9736	5.0118
Cefazolin	4.2439	4.2298	4.2581
Methylprednisolone	3.8684	3.8541	3.8824
Ranitidine	3.8649	3.8462	3.8827
Diphenhydramine	3.5479	3.5310	3.5636
Lidocaine prilocaine	3.4114	3.3841	3.4403
Dexamethasone	3.0949	3.0809	3.1102
Promethazine	2.8213	2.8067	2.8358
Metoclopramide	2.7527	2.7382	2.7673
Ketorolac	2.7080	2.6939	2.7214
Cefotaxime	2.6960	2.6794	2.7125
Calcium gluconate	2.6932	2.6770	2.7094
Prednisolone	2.6815	2.6673	2.6951
Ipratropium	2.5705	2.5572	2.5831
Vancomycin	2.5602	2.5477	2.5719
Rocuronium	2.5251	2.5126	2.5374
Bacitracin	2.4278	2.4075	2.4478
Clindamycin	2.4258	2.4135	2.4392
Glycopyrrolate	2.3598	2.3481	2.3723
Levalbuterol	2.3251	2.3116	2.3392
Acetaminophen codeine	2.2798	2.2662	2.2927
Azithromycin	2.2722	2.2584	2.2849
Furosemide	2.2063	2.1954	2.2167
Bupivacaine	2.1625	2.1496	2.1760
Lorazepam	2.1326	2.1197	2.1442
Oxytocin	2.1101	2.1015	2.1189
Docusate	2.0829	2.0714	2.0954
Sodium bicarbonate	1.9117	1.8987	1.9247
Famotidine	1.9043	1.8932	1.9164
Hydrocodone acetaminophen	1.9021	1.8901	1.9140
Epinephrine	1.8896	1.8769	1.9019
Neostigmine	1.8181	1.8076	1.8292
Magnesium	1.8079	1.7966	1.8191
Budesonide	1.7875	1.7753	1.7991
Nystatin	1.7684	1.7551	1.7821
Meperidine	1.5186	1.5078	1.5304
Succinylcholine chloride	1.4648	1.4533	1.4755
Glycerin supplement	1.4441	1.4318	1.4563
Oxycodone asprin	1.4261	1.4139	1.4381
Montelukast	1.3879	1.3774	1.3988
Bupivacaine epinephrine	1.3571	1.3465	1.3673

Iron sulfate	1.3472	1.3378	1.3572
Lansoprazole	1.3459	1.3352	1.3567
Trimethoprim sulfamethoxazole	1.2482	1.2382	1.2578
Atropine	1.2013	1.1923	1.2104
Fluticasone	1.1534	1.1438	1.1633
Polyethylene glycol electrolyte	1.1256	1.1160	1.1358
Vecuronium	1.1062	1.0966	1.1155
Lidocaine cardiac	1.0816	1.0672	1.0951
Simethicone	1.0598	1.0476	1.0718
Albumin	1.0432	1.0355	1.0514
Metronidazole	0.9892	0.9804	0.9980
Lidocaine epinephrine	0.9888	0.9800	0.9975
Hydromorphone	0.9887	0.9798	0.9971
Ampicillin sulbactam	0.9867	0.9763	0.9967
Immune globulin hepatitis b	0.9741	0.9524	0.9979
Dopamine	0.9528	0.9459	0.9602
Prednisone	0.9471	0.9391	0.9554
Amoxicillin	0.9451	0.9357	0.9549
Phenylephrine	0.9449	0.9358	0.9540
Potassium phosphate	0.9427	0.9344	0.9508
Insulin	0.9112	0.9047	0.9177
Caffeine	0.9054	0.8984	0.9122
Palivizumab	0.9043	0.8965	0.9126
Hydrocortisone	0.8661	0.8571	0.8749
Ioversol	0.8322	0.8238	0.8417
Phenobarbital	0.8253	0.8168	0.8332
Benzocaine	0.8149	0.8071	0.8228
Cefuroxime	0.8088	0.8004	0.8175
Dolasetron	0.8004	0.7927	0.8084
Piperacillin tazobactam	0.7983	0.7900	0.8060
Neomycin polymixin bacitracin	0.7785	0.7628	0.7949
Mupirocin	0.7781	0.7679	0.7886
Butorphanol	0.7702	0.7630	0.7776
Bisacodyl	0.7536	0.7459	0.7628
Chloral	0.7309	0.7232	0.7388
Amoxicillin clavulanate	0.7150	0.7066	0.7230
Potassium acetate	0.7057	0.6972	0.7139
Sodium acetate	0.7036	0.6963	0.7114
Cefoxitin	0.6913	0.6848	0.6985
Fluconazole	0.6871	0.6797	0.6943
Oxycodone	0.6656	0.6546	0.6768
Pantoprazole	0.6516	0.6444	0.6596
Acyclovir	0.6514	0.6431	0.6597
Thrombin	0.6414	0.6353	0.6475
Naloxone	0.6364	0.6235	0.6481
Aluminum magnesium hydroxide	0.6240	0.6144	0.6343
Diazepam	0.6160	0.6086	0.6231
Ketamine	0.6112	0.6041	0.6188
Cyclopentolate phenylephrine	0.6055	0.5991	0.6118
Nalbuphine	0.5919	0.5843	0.6004
Hydroxyzine	0.5898	0.5830	0.5974
Polymyxin bacitracin	0.5896	0.5740	0.6057
Calcium chloride	0.5885	0.5825	0.5941
Ceftazidime	0.5675	0.5605	0.5744
Cefepime	0.5625	0.5563	0.5697

Penicillin G	0.5604	0.5520	0.5685
L-cysteine	0.5114	0.5045	0.5179
Magnesium hydroxide	0.5059	0.4995	0.5122
Risperidone	0.4903	0.4844	0.4970
Tobramycin	0.4897	0.4827	0.4973
Sodium phosphate	0.4896	0.4830	0.4959
Beractant	0.4884	0.4818	0.4950
Ropivacaine	0.4723	0.4661	0.4791
Aspirin	0.4641	0.4583	0.4702
Quetiapine fumarate	0.4544	0.4485	0.4603
Fluoxetine	0.4362	0.4308	0.4416
Sodium citrate	0.4290	0.4232	0.4349
Ephedrine	0.4288	0.4225	0.4347
Divalproex sodium	0.4258	0.4204	0.4316
Chlorhexidine	0.4213	0.4157	0.4275
Oxcarbazepine	0.4198	0.4138	0.4263
Mannitol	0.4196	0.4144	0.4247
Zolpidem	0.4169	0.4107	0.4231
Clonidine	0.4157	0.4098	0.4216
Vaccine: pneumococcal	0.4110	0.4056	0.4167
Cetirizine	0.3999	0.3943	0.4061
Fentanyl bupivacaine	0.3988	0.3919	0.4054
Ciprofloxacin	0.3883	0.3824	0.3949
Fosphenytoin	0.3859	0.3803	0.3912
Mivacurium	0.3849	0.3788	0.3911
Vitamin B9	0.3827	0.3773	0.3881
Alteplase	0.3818	0.3765	0.3870
Lactobacillus	0.3785	0.3724	0.3845
Senna	0.3761	0.3704	0.3824
Lanolin	0.3715	0.3626	0.3805
Cephalexin	0.3699	0.3641	0.3759
Oxymetazoline	0.3675	0.3619	0.3730
Epoetin	0.3670	0.3616	0.3725
Levetiracetam	0.3640	0.3585	0.3694
Senna docusate	0.3596	0.3538	0.3657
Sertraline	0.3556	0.3497	0.3614
Aripiprazole	0.3525	0.3474	0.3575
Loratadine	0.3524	0.3469	0.3578
Immune globulin human ig	0.3512	0.3458	0.3568
Escitalopram oxalate	0.3452	0.3396	0.3506
Poractant	0.3449	0.3390	0.3508
Zinc	0.3416	0.3358	0.3471
Indomethacin	0.3394	0.3347	0.3443
Milrinone	0.3376	0.3339	0.3415
Propoxyphene acetaminophen	0.3345	0.3292	0.3403
Levocarnitine	0.3299	0.3246	0.3351
Fluticasone salmeterol	0.3252	0.3193	0.3304
Spironolactone	0.3215	0.3167	0.3265
Ipratropium albuterol	0.3170	0.3115	0.3222
Granisetron	0.3124	0.3069	0.3184
Topiramate	0.3117	0.3063	0.3169
Gadopentetate	0.3100	0.3045	0.3158
Digoxin	0.3024	0.2976	0.3075
Pancuronium	0.3023	0.2972	0.3075
Cisatracurium	0.3019	0.2962	0.3071

Levofloxacin	0.2998	0.2949	0.3049
Enoxaparin	0.2993	0.2944	0.3044
Phenytoin	0.2990	0.2940	0.3043
Filgrastim	0.2961	0.2912	0.3004
Valproic acid	0.2926	0.2875	0.2978
Levothyroxine	0.2898	0.2847	0.2952
Methylphenidate	0.2887	0.2841	0.2940
Vincristine	0.2871	0.2823	0.2916
Physostigmine	0.2827	0.2705	0.2949
Nafcillin	0.2814	0.2761	0.2863
Dornase	0.2793	0.2751	0.2833
Iron sucrose	0.2792	0.2678	0.2912
Protriptyline	0.2748	0.2630	0.2870
Vaccine: influenza	0.2736	0.2686	0.2788
Trazodone	0.2732	0.2684	0.2780
Enalapril	0.2720	0.2674	0.2772
Calcium carbonate	0.2691	0.2631	0.2741
Cefdinir	0.2670	0.2620	0.2727
Vaccine: haemophilus b	0.2667	0.2623	0.2710
Mesna	0.2663	0.2622	0.2702
Lamotrigine	0.2658	0.2612	0.2710
Calfactant	0.2626	0.2579	0.2675
Diatrizoate meglumine and diatrizoate sodium	0.2578	0.2533	0.2624
Chlorothiazide	0.2573	0.2529	0.2618
Atracurium	0.2565	0.2520	0.2607
Pentobarbital	0.2541	0.2492	0.2591
Meropenem	0.2533	0.2486	0.2580
Clonazepam	0.2531	0.2486	0.2577
Methotrexate	0.2510	0.2460	0.2553
Sucralfate	0.2498	0.2450	0.2548
Cyclophosphamide	0.2462	0.2425	0.2500
Guaifenesin	0.2439	0.2390	0.2489
Hydrocortisone sodium succinate	0.2438	0.2393	0.2486
Terbutaline	0.2410	0.2363	0.2459
Dobutamine	0.2408	0.2365	0.2451
Omeprazole	0.2379	0.2332	0.2431
Acetylcysteine	0.2376	0.2328	0.2426
Ferrous sulfate	0.2376	0.2323	0.2423
Ursodiol	0.2372	0.2327	0.2416
Methadone	0.2370	0.2325	0.2414
Pramoxine	0.2356	0.2309	0.2403
Triamcinolone	0.2345	0.2298	0.2392
Sevoflurane	0.2334	0.2284	0.2383
Mometasone	0.2302	0.2253	0.2349
Silver sulfadiazine	0.2268	0.2227	0.2308
Labetalol	0.2220	0.2173	0.2266
Trimethobenzamide	0.2117	0.2071	0.2161
Carbamazepine	0.2077	0.2034	0.2124
Clotrimazole	0.2030	0.1987	0.2076
Oxacillin	0.2028	0.1986	0.2071
Vitamin D	0.2026	0.1983	0.2068
Dinoprostone	0.2008	0.1964	0.2047
Tetracaine	0.2000	0.1960	0.2040
Papaverine	0.1990	0.1949	0.2030
Baclofen	0.1983	0.1940	0.2026

Tropicamide	0.1975	0.1930	0.2019
Etoposide	0.1964	0.1927	0.2000
Vaccine: diptheria, tetanus	0.1912	0.1872	0.1951
Misoprostol	0.1910	0.1867	0.1954
Oxybutynin	0.1872	0.1837	0.1908
Medroxyprogesterone	0.1869	0.1828	0.1908
Cytarabine	0.1868	0.1828	0.1903
Dextroamphetamine amphetamine	0.1841	0.1803	0.1880
Nitroprusside	0.1820	0.1785	0.1852
Naproxen	0.1802	0.1761	0.1845
Olanzapine	0.1792	0.1753	0.1832
Etomidate	0.1785	0.1746	0.1824
Povidone iodine	0.1776	0.1735	0.1818
Gadolinium	0.1770	0.1727	0.1808
Benztropine	0.1761	0.1712	0.1807
Esomeprazole	0.1757	0.1716	0.1795
Amlodipine	0.1750	0.1712	0.1789
Nifedipine	0.1743	0.1702	0.1781
Methylergon	0.1727	0.1681	0.1769
Iohexol	0.1725	0.1685	0.1765
Vaccine: diptheria, tetanus, pertussis, hepatits b, polio	0.1706	0.1671	0.1744
Haloperidol	0.1698	0.1657	0.1739
Vaccine: haemophilus b, hepatitis b	0.1685	0.1599	0.1762
Ticarcillin clavulanate	0.1683	0.1642	0.1724
Cefotetan	0.1673	0.1633	0.1715
Gabapentin	0.1647	0.1607	0.1691
Selenium	0.1645	0.1600	0.1690
Silver nitrate	0.1641	0.1570	0.1718
Bupropion	0.1636	0.1598	0.1672
Lactulose	0.1634	0.1596	0.1673
Vaccine: diptheria, tetanus, pertussis	0.1624	0.1585	0.1661
Sufentanil	0.1612	0.1575	0.1651
Ziprasidone	0.1597	0.1560	0.1634
Neomycin polymyxin	0.1584	0.1541	0.1629
Doxycycline	0.1583	0.1546	0.1620
Guaifenesin dextromethorphan	0.1579	0.1540	0.1620
Amphotericin b	0.1577	0.1539	0.1615
Captopril	0.1575	0.1541	0.1610
Leucovorin	0.1572	0.1537	0.1609
Thiopental	0.1565	0.1526	0.1606
Aprotinin	0.1555	0.1527	0.1583
Immune globulin rho d	0.1540	0.1506	0.1579
Aminophylline	0.1536	0.1497	0.1574
Prochlorperazine	0.1510	0.1472	0.1548
Cyclopentolate	0.1498	0.1462	0.1538
Penicillin v	0.1479	0.1442	0.1517
Vaccine: polio	0.1465	0.1431	0.1499
Desmopressin	0.1451	0.1414	0.1488
Acetazolamide	0.1437	0.1403	0.1475
Doxorubicin	0.1437	0.1405	0.1470
Alprostadil	0.1436	0.1405	0.1470
Vitamin a	0.1410	0.1375	0.1445
Hydralazine	0.1366	0.1331	0.1399
Betamethasone	0.1348	0.1316	0.1377

Propranolol	0.1330	0.1294	0.1365
Remifentanil	0.1321	0.1288	0.1355
Pseudoephedrine	0.1316	0.1283	0.1353
Ofloxacin	0.1301	0.1268	0.1336
Lithium	0.1299	0.1268	0.1334
Rifampin	0.1295	0.1261	0.1328
Atomoxetine	0.1259	0.1223	0.1291
Phenol	0.1257	0.1221	0.1287
Proparacaine	0.1250	0.1219	0.1284
Vaccine: rubella	0.1250	0.1215	0.1286
Tacrolimus	0.1235	0.1201	0.1267
Zolmitriptan	0.1223	0.1190	0.1257
Protamine sulfate	0.1222	0.1197	0.1246
Iodixanol	0.1221	0.1189	0.1253
Methylene blue	0.1219	0.1189	0.1248
Nitroglycerin	0.1198	0.1166	0.1234
Benzoin tincture	0.1183	0.1150	0.1219
Kanamycin	0.1172	0.1139	0.1206
Vitamin e	0.1142	0.1109	0.1174
Mercaptopurine	0.1134	0.1098	0.1166
Loperamide	0.1130	0.1097	0.1162
Dexmedetomidine	0.1123	0.1093	0.1151
Protamine	0.1108	0.1085	0.1131
Technetium-99m unspecified	0.1105	0.1072	0.1139
Vitamin c	0.1096	0.1063	0.1127
Clarithromycin	0.1082	0.1049	0.1115
Theophylline	0.1044	0.1013	0.1074
Codeine	0.1039	0.1005	0.1072
Nicotine	0.1037	0.1006	0.1070
Edrophonium	0.1032	0.1003	0.1065
Linezolid	0.1021	0.0992	0.1052
Oseltamivir	0.1006	0.0978	0.1035
Adenosine	0.1002	0.0972	0.1037
Mycophenolate	0.0999	0.0970	0.1025
Cholestyramine light	0.0995	0.0964	0.1028
Amikacin	0.0988	0.0955	0.1019
Imipenem	0.0976	0.0946	0.1008
Miconazole	0.0974	0.0939	0.1003
Salmeterol xinafoate fluticasone	0.0922	0.0892	0.0953
Droperidol	0.0910	0.0879	0.0937
Chlorpromazine	0.0905	0.0875	0.0935
Ifosfamide	0.0889	0.0861	0.0915
Cisplatin	0.0879	0.0853	0.0905
Moxifloxacin	0.0864	0.0833	0.0898
Cyproheptadine	0.0860	0.0831	0.0888
Citalopram	0.0831	0.0803	0.0860
Venlafaxine	0.0831	0.0805	0.0858
Allopurinol	0.0827	0.0804	0.0848
Warfarin	0.0816	0.0789	0.0841
Mepivacaine	0.0814	0.0784	0.0847
Esmolol	0.0811	0.0783	0.0836
Barium sulfate	0.0807	0.0777	0.0836
Amitriptyline	0.0803	0.0776	0.0830
Chloroprocaine	0.0803	0.0746	0.0856
Darbepoetin albumin	0.0803	0.0777	0.0831

Ertapenem	0.0800	0.0774	0.0827
Iron	0.0799	0.0772	0.0826
Tromethamine	0.0799	0.0769	0.0827
Hyoscyamine	0.0798	0.0772	0.0824
Flumazenil	0.0795	0.0766	0.0822
Magnesia	0.0792	0.0763	0.0821
Dibucaine	0.0791	0.0756	0.0823
Chloroethane	0.0786	0.0759	0.0816
Mesalamine	0.0783	0.0762	0.0806
Norepinephrine	0.0778	0.0753	0.0803
Polymyxin trimethoprim	0.0771	0.0736	0.0807
Scopolamine	0.0770	0.0741	0.0795
Nitrofuran	0.0766	0.0738	0.0791
Cyclosporine	0.0748	0.0724	0.0773
Polymyxin	0.0746	0.0720	0.0772
Cefprozil	0.0742	0.0714	0.0768
Atenolol	0.0740	0.0715	0.0765
Isoflurane	0.0739	0.0714	0.0767
Aminocaproic acid	0.0738	0.0715	0.0760
Pentamidine	0.0733	0.0707	0.0758
Beclomethasone	0.0731	0.0705	0.0759
Physiosol	0.0730	0.0705	0.0753
Pancrelipase	0.0719	0.0697	0.0742
Cyclobenzaprine	0.0716	0.0692	0.0742
Flunisolide	0.0713	0.0688	0.0738
Paroxetine	0.0713	0.0685	0.0741
Magnesium carbonate	0.0712	0.0686	0.0739
Iopamidol	0.0711	0.0686	0.0737
Diatrizoate	0.0705	0.0679	0.0731
Hydroxyurea	0.0701	0.0676	0.0725
Mirtazapine	0.0701	0.0676	0.0727
Lisinopril	0.0698	0.0671	0.0723
Hyaluronidase	0.0689	0.0663	0.0715
Iothalamate	0.0689	0.0664	0.0713
Ticarcillin	0.0680	0.0660	0.0710
Metoprolol	0.0678	0.0653	0.0703
Calamine pramoxine	0.0671	0.0636	0.0708
Pseudoephedrine carbinoxamine dextromethorphan	0.0669	0.0645	0.0695
Zidovudine	0.0663	0.0615	0.0710
Gentian violet	0.0657	0.0603	0.0713
Voriconazole	0.0648	0.0625	0.0673
Nystatin triamcinolone	0.0645	0.0621	0.0671
Metformin	0.0642	0.0617	0.0668
Cimetidine	0.0637	0.0611	0.0664
Bumetanide	0.0631	0.0606	0.0654
Metolazone	0.0628	0.0605	0.0652
Phenazopyridine	0.0623	0.0598	0.0648
Glucagon	0.0620	0.0593	0.0645
Fexofenadine	0.0603	0.0579	0.0626
Vasopressin	0.0603	0.0581	0.0626
Iron fumarate docusate sodium	0.0599	0.0574	0.0623
Pseudoephedrine brompheniramine	0.0597	0.0574	0.0623
Nicardipine	0.0591	0.0569	0.0615
Sodium lactate	0.0582	0.0555	0.0610

Hespan	0.0574	0.0551	0.0596
Pegaspargase	0.0572	0.0553	0.0593
Vitamin b6	0.0559	0.0538	0.0581
Neomycin	0.0541	0.0512	0.0573
Tramadol	0.0537	0.0515	0.0564
Duloxetine	0.0526	0.0503	0.0548
Dapsone	0.0523	0.0501	0.0545
Vaccine: measles, mumps, rubella	0.0521	0.0499	0.0543
Docusate sodium casanthranol*	0.0519	0.0499	0.0543
Amiodarone	0.0517	0.0495	0.0539
Guanfacine	0.0517	0.0495	0.0541
Cefaclor	0.0500	0.0469	0.0533
Azathioprine	0.0498	0.0478	0.0519
Alprazolam	0.0476	0.0455	0.0496
Sulfacetamide op	0.0472	0.0446	0.0498
Ciprofloxacin dexameth otic	0.0468	0.0449	0.0490
Carboprost tromethamine	0.0467	0.0445	0.0488
Cosyntropin	0.0463	0.0442	0.0486
Tobramycin dexamethasone opthalamic	0.0463	0.0440	0.0488
Vinblastine	0.0463	0.0442	0.0484
Temazepam	0.0451	0.0430	0.0470
Vitamin b1	0.0451	0.0431	0.0472
Daunorubicin	0.0450	0.0431	0.0469
Hydrochlorothiazide	0.0445	0.0424	0.0465
Psyllium	0.0441	0.0420	0.0462
Dicyclomine	0.0440	0.0419	0.0462
Megestrol	0.0434	0.0413	0.0455
Vaccine: tetanus	0.0432	0.0412	0.0453
Ammonium chloride	0.0426	0.0405	0.0445
Vacc dipth teta pertussis polio	0.0416	0.0367	0.0463
Peptamen	0.0415	0.0397	0.0433
Guaifenesin codeine	0.0409	0.0391	0.0428
Neomycin polymyxin hydrocortisone	0.0406	0.0386	0.0426
Permethrin	0.0406	0.0386	0.0426
Vitamin B12	0.0405	0.0386	0.0427
Dextroamphet	0.0401	0.0382	0.0418
Factor viia	0.0399	0.0381	0.0419
Carboplatin	0.0393	0.0373	0.0413
Fludrocortisone	0.0388	0.0369	0.0407
Valganciclovir	0.0380	0.0364	0.0396
Caspofungin	0.0372	0.0354	0.0391
Aztreonam	0.0370	0.0352	0.0389
Dactinomycin	0.0367	0.0350	0.0383
Hydroxychloroquine	0.0349	0.0332	0.0366
Dexamethasone neomycin	0.0345	0.0327	0.0362
Asparaginase	0.0340	0.0323	0.0360
Quetiapine	0.0339	0.0321	0.0357
Diphenoxylate atropine	0.0336	0.0318	0.0353
Sildenafil	0.0332	0.0312	0.0350
Ganciclovir	0.0330	0.0315	0.0345
Irinotecan	0.0327	0.0312	0.0345
Dexmethylphenidate	0.0324	0.0307	0.0340
Iron gluconate	0.0322	0.0306	0.0339
Celecoxib	0.0316	0.0300	0.0333

Phenylpropanolamine chlorpheniramine dextromethorphan	0.0315	0.0299	0.0333
Calcium acetate	0.0314	0.0296	0.0332
Octreotide	0.0312	0.0295	0.0329
Dihydroergotamine	0.0311	0.0297	0.0326
Hydrocortisone polyethylene glycol	0.0306	0.0290	0.0322
Buspirone	0.0304	0.0287	0.0319
Isoproterenol	0.0303	0.0287	0.0319
Dextran	0.0300	0.0283	0.0319
Dronabinol	0.0294	0.0277	0.0310
Bleomycin	0.0285	0.0268	0.0301
Clorazepate	0.0281	0.0265	0.0298
Cromolyn	0.0277	0.0259	0.0293
Imipramine	0.0277	0.0259	0.0295
Nitrofurantoin	0.0276	0.0259	0.0292
Modafinil	0.0275	0.0260	0.0292
Cefpodoxime	0.0273	0.0257	0.0290
Ceftizoxime	0.0269	0.0253	0.0285
Nitrousoxide	0.0268	0.0251	0.0285
Pegfilgrastim	0.0267	0.0251	0.0282
Ketoconazole	0.0266	0.0250	0.0282
Donnatal	0.0260	0.0245	0.0276
Tizanidine	0.0259	0.0243	0.0275
Ciprofloxacin hc otic	0.0258	0.0243	0.0275
Methocarbamol	0.0258	0.0243	0.0274
Metaproterenol	0.0256	0.0238	0.0276
Alfentanil	0.0252	0.0237	0.0268
Glutamine	0.0249	0.0232	0.0263
Thioguanine	0.0249	0.0233	0.0264
Gatifloxacin	0.0238	0.0223	0.0253
Bethanechol	0.0237	0.0223	0.0254
Valacyclovir	0.0236	0.0221	0.0252
Promethazine codeine	0.0235	0.0219	0.0250
Benzonatate	0.0230	0.0215	0.0245
Sumatriptan	0.0230	0.0216	0.0244
Sirolimus	0.0225	0.0210	0.0239
Infliximab	0.0224	0.0209	0.0238
Carbamide peroxide otic	0.0223	0.0207	0.0240
Ethacrynic acid	0.0223	0.0208	0.0237
Aprepitant	0.0222	0.0208	0.0237
Erythromycin sulfisoxazole	0.0222	0.0207	0.0236
Amphetamine complex	0.0221	0.0206	0.0237
Piperacillin	0.0221	0.0207	0.0236
Felbamate	0.0219	0.0205	0.0235
Pimecrolimus	0.0217	0.0202	0.0231
Minocycline	0.0215	0.0202	0.0230
Isradipine	0.0212	0.0198	0.0227
Estrogen conjugated	0.0209	0.0195	0.0224
Arginine	0.0208	0.0194	0.0221
Verapamil	0.0208	0.0194	0.0221
Caffeine sodium benzoate	0.0207	0.0195	0.0220
Sincalide	0.0205	0.0192	0.0219
Phenylpropanolamine brompheniramine	0.0204	0.0192	0.0218
Cephradine	0.0199	0.0188	0.0210
Sodium ferric gluconate complex	0.0195	0.0181	0.0208

Aspirin codeine	0.0193	0.0164	0.0223
Phentolamine	0.0193	0.0179	0.0208
Phenylephrine chlorpheniramine hydrocodone	0.0192	0.0178	0.0206
Iothalamate meglumine	0.0191	0.0176	0.0205
Metrizamide	0.0191	0.0176	0.0206
Isoniazid	0.0190	0.0175	0.0205
Oxymorphone	0.0190	0.0176	0.0204
Dextromethorphan	0.0188	0.0176	0.0202
Rituximab	0.0188	0.0175	0.0201
Hydrocodone chlorpheniramine	0.0185	0.0171	0.0197
Tegaserod	0.0182	0.0170	0.0196
Cocaine	0.0181	0.0167	0.0194
Dialysis Solution	0.0180	0.0170	0.0190
Edrophonium atropine	0.0179	0.0166	0.0192
Meperidine promethazine	0.0179	0.0167	0.0193
Pseudoephedrine carbinoxamine	0.0179	0.0167	0.0192
Tolterodine	0.0179	0.0166	0.0192
Corticotropin	0.0178	0.0165	0.0190
Diltiazem	0.0176	0.0164	0.0189
Fluvoxamine	0.0176	0.0163	0.0189
Opium	0.0176	0.0163	0.0191
Cholestyramine	0.0175	0.0162	0.0188
Lidocaine epinepherine tetracaine	0.0172	0.0160	0.0185
Cefixime	0.0168	0.0155	0.0183
Diatrizoate sodium	0.0164	0.0150	0.0179
Nitazoxanide	0.0163	0.0152	0.0175
Carvedilol	0.0160	0.0148	0.0174
Belladonna opium	0.0159	0.0146	0.0171
Itraconazole	0.0158	0.0145	0.0170
Sevelamer	0.0158	0.0147	0.0170
Ferric subsulfate solution	0.0157	0.0143	0.0172
Guaifenesin pseudoephedrine	0.0156	0.0144	0.0168
Prazosin	0.0155	0.0143	0.0168
Pseudoephedrine brompheniramine dextromethorphan	0.0152	0.0140	0.0165
Tetracycline	0.0152	0.0140	0.0164
Undecylenic acid	0.0152	0.0135	0.0171
Pyridostigmine	0.0146	0.0135	0.0158
Aluminum acetate	0.0145	0.0134	0.0157
Citrate phosphate dextr	0.0144	0.0132	0.0156
Desflurane	0.0143	0.0131	0.0155
Mitoxantrone	0.0143	0.0132	0.0156
Acetaminophen caffeine butalbital	0.0141	0.0130	0.0154
Dalteparin	0.0141	0.0130	0.0152
Formoterol fumarate	0.0141	0.0130	0.0153
Tamsulosin	0.0141	0.0130	0.0151
Gadodiamide	0.0139	0.0128	0.0151
Dantrolene	0.0138	0.0126	0.0150
Guaifenesin	0.0138	0.0126	0.0150
Methyoxyflurane	0.0137	0.0125	0.0150
Amantadine	0.0135	0.0124	0.0147
Atovaquone	0.0135	0.0124	0.0146
Indigotindisulfonate	0.0135	0.0124	0.0146
Vaccine: meningococcal	0.0135	0.0124	0.0146
Loratadine pseudoephedrine	0.0131	0.0121	0.0142

Leuprolide depot	0.0130	0.0118	0.0141
Reteplase	0.0130	0.0120	0.0140
Factor viii	0.0129	0.0118	0.0142
Rasburicase	0.0129	0.0119	0.0140
Pregabalin	0.0128	0.0117	0.0138
Hydrocortisone sodium phosphate	0.0126	0.0115	0.0137
Pramipexole	0.0126	0.0115	0.0136
Nortriptyline	0.0124	0.0115	0.0136
Dimenhydrinate	0.0123	0.0112	0.0134
Propoxyphene	0.0121	0.0110	0.0132
Desloratadine	0.0119	0.0108	0.0130
Fluorouracil	0.0119	0.0109	0.0130
Iodine	0.0118	0.0106	0.0130
Methyldopa	0.0118	0.0107	0.0130
Fluorescein	0.0117	0.0107	0.0129
Hydrocodone guaifenesin	0.0117	0.0107	0.0128
Sodium thiosulfate	0.0115	0.0104	0.0125
Antithymocyte globulin	0.0114	0.0105	0.0125
Brompheniramine	0.0114	0.0104	0.0125
Gadodiamide caldiamide	0.0113	0.0103	0.0122
Sotalol	0.0111	0.0102	0.0121
Dexrazoxane	0.0110	0.0100	0.0120
Lomustine	0.0110	0.0100	0.0120
Orphenadrine	0.0110	0.0100	0.0119
Melatonin	0.0109	0.0099	0.0119
L hyoscyamine sulfate	0.0106	0.0095	0.0116
Pamidronate	0.0106	0.0096	0.0116
Atorvastatin	0.0105	0.0095	0.0116
Fluocinonide	0.0105	0.0094	0.0114
Vaccine: hepatitis a	0.0105	0.0089	0.0120
Somatropin	0.0102	0.0092	0.0112
Acetaminophen chlorpheniramine dextromethorphan pseudoephedrine	0.0101	0.0091	0.0111
Cefamandole	0.0099	0.0089	0.0109
Sulfasalazine	0.0098	0.0088	0.0108
Glyburide	0.0097	0.0088	0.0107
Liothyronine	0.0097	0.0086	0.0106
Vaccine: varicella	0.0097	0.0087	0.0106
Busulfan	0.0096	0.0089	0.0104
Collagenase	0.0096	0.0087	0.0106
Daclizumab	0.0096	0.0088	0.0104
Albuterol ipratropium	0.0094	0.0084	0.0104
Cefadroxil	0.0094	0.0086	0.0103
Glutamic acid hcl	0.0094	0.0085	0.0103
Hyaluronate	0.0093	0.0084	0.0102
Imipenem cilastatin	0.0092	0.0083	0.0102
Lamivudine	0.0092	0.0082	0.0102
Minoxidil	0.0092	0.0083	0.0102
Vaccine: diptheria, tetanus, pertussis, haemophilus b	0.0092	0.0082	0.0102
Dipyridamole	0.0090	0.0080	0.0101
Fluoride	0.0090	0.0081	0.0098
Carbamide	0.0089	0.0079	0.0100
Mitomycin	0.0089	0.0081	0.0098

Azelastine	0.0088	0.0079	0.0098
Mafenide	0.0088	0.0081	0.0097
Deferoxamine	0.0087	0.0078	0.0096
Ethambutol	0.0086	0.0077	0.0095
Diazoxide	0.0085	0.0074	0.0096
Drotrecogin alfa	0.0084	0.0075	0.0094
Clopidogrel	0.0083	0.0074	0.0093
Doxapram	0.0082	0.0073	0.0090
Flecainide	0.0082	0.0074	0.0091
Pyrazinamide	0.0082	0.0073	0.0091
Salmeterol	0.0081	0.0073	0.0091
Meclizine	0.0080	0.0072	0.0089
Polycarbophil	0.0080	0.0071	0.0089
Nalmefene	0.0079	0.0069	0.0089
Temozolomide	0.0079	0.0070	0.0088
Argatroban	0.0078	0.0069	0.0088
Charcoal	0.0078	0.0070	0.0087
Immune globulin varicella zoster	0.0078	0.0069	0.0086
Fludarabine	0.0076	0.0067	0.0084
Trihexyphenidyl	0.0076	0.0067	0.0085
Darbepoetin alfa	0.0074	0.0065	0.0083
Lopinavir ritonavir	0.0074	0.0067	0.0081
Urokinase	0.0074	0.0066	0.0083
Botulinum toxin type a	0.0073	0.0065	0.0082
Lindane	0.0073	0.0066	0.0082
Tiagabine	0.0073	0.0065	0.0081
Topotecan hcl	0.0070	0.0061	0.0078
Homatropine	0.0069	0.0061	0.0076
Micafungin	0.0069	0.0060	0.0077
Aluminum	0.0068	0.0060	0.0077
Bromocriptine	0.0067	0.0058	0.0075
Losartan	0.0066	0.0057	0.0075
Potassium iodide	0.0066	0.0058	0.0074
Ethosuximide	0.0065	0.0057	0.0074
Melphalan	0.0064	0.0057	0.0070
Olopatadine	0.0064	0.0056	0.0071
Fluocinolone	0.0062	0.0055	0.0070
Primidone	0.0062	0.0055	0.0070
Immune globulin cmv	0.0061	0.0053	0.0069
Tranexamic acid	0.0061	0.0053	0.0070
Colistimethate	0.0060	0.0052	0.0068
Sargramostim	0.0060	0.0052	0.0068
Trifluridine	0.0060	0.0050	0.0069
Doxazosin	0.0058	0.0049	0.0067
Methohexital	0.0058	0.0050	0.0065
Procainamide	0.0057	0.0050	0.0066
Dorzolamide	0.0055	0.0048	0.0063
Latanoprost	0.0055	0.0047	0.0062
Perphenazine	0.0055	0.0048	0.0063
Valsartan	0.0055	0.0048	0.0062
Nadolol	0.0053	0.0047	0.0061
Basiliximab	0.0052	0.0047	0.0058
Nesiritide	0.0052	0.0045	0.0058
Factor ix	0.0050	0.0044	0.0057
Epoprostenol	0.0049	0.0043	0.0056

Fenoldopam	0.0049	0.0042	0.0056
Gemtuzumab ozogamicin	0.0049	0.0042	0.0056
Vitamin b2	0.0049	0.0041	0.0056
Carbidopa levodopa	0.0048	0.0041	0.0055
Cladribine	0.0048	0.0041	0.0055
Dicloxacillin	0.0048	0.0041	0.0056
Griseofulvin	0.0048	0.0041	0.0056
Zalepon	0.0048	0.0042	0.0055
Dorzolamide timolol	0.0046	0.0040	0.0053
Doxepin	0.0046	0.0039	0.0052
Lymphocyte immune globulin	0.0046	0.0039	0.0053
Alemtuzumab	0.0044	0.0038	0.0051
Alendronate	0.0044	0.0038	0.0051
Clomipramine	0.0043	0.0037	0.0049
Ioxaglate meglumine, ioxaglate sodium	0.0043	0.0036	0.0050
Nevirapine	0.0040	0.0030	0.0052
Methimazole	0.0039	0.0033	0.0045
Ritonavir	0.0038	0.0033	0.0043
Rosiglitazone	0.0038	0.0032	0.0045
Zanamivir	0.0037	0.0030	0.0043
Imatinib mesylate	0.0036	0.0030	0.0042
Fludeoxyglucose	0.0035	0.0029	0.0041
Human antithrombin iii	0.0034	0.0027	0.0040
Gadoteridol	0.0034	0.0028	0.0040
Balsalazide	0.0033	0.0027	0.0039
Probenecid	0.0032	0.0026	0.0038
Leuprolide	0.0031	0.0025	0.0036
Cidofovir	0.0030	0.0025	0.0036
Potassium phosphate sodium phosphate	0.0030	0.0024	0.0036
Sodium sulfacetamide	0.0030	0.0025	0.0036
Stavudine	0.0030	0.0025	0.0035
Amiloride	0.0029	0.0024	0.0035
Estradiol	0.0029	0.0024	0.0034
Pravastatin	0.0029	0.0024	0.0035
Cisapride	0.0028	0.0023	0.0034
Palonosetron hcl	0.0028	0.0022	0.0033
Chromium	0.0027	0.0022	0.0033
Edetate calcium disodium	0.0027	0.0022	0.0033
Thiotepa	0.0027	0.0022	0.0032
Vaccine: rabies	0.0027	0.0021	0.0033
Ribavirin	0.0024	0.0020	0.0029
Flucytosine	0.0023	0.0018	0.0028
Tretinoin	0.0023	0.0018	0.0028
Trimethoprim	0.0023	0.0019	0.0029
Foscarnet	0.0022	0.0017	0.0027
Idarubicin	0.0020	0.0016	0.0024
Iodine 131	0.0020	0.0015	0.0024
Timolol	0.0020	0.0016	0.0025
Halothane	0.0019	0.0014	0.0024
Mexiletine	0.0019	0.0014	0.0023
Procarbazine	0.0019	0.0015	0.0023
Bosentan	0.0018	0.0013	0.0023
Formaldehyde	0.0018	0.0013	0.0023
Promazine	0.0018	0.0014	0.0022
Succimer	0.0016	0.0013	0.0020

Interferon gamma 1b	0.0013	0.0010	0.0017
Sodium phenylbutyrate	0.0013	0.0010	0.0017
Sodium benzoate	0.0012	0.0009	0.0015
Testosterone	0.0012	0.0009	0.0016
Norethindrone	0.0011	0.0008	0.0014
Dextroamphetamin sulfate	0.0010	0.0007	0.0014

Table B: Estimated Percentage of Patients Exposed to the 700 Most Commonly Used Medications, Sorted Alphabetically

Drug	Estimate	95% CI LL	95% CI UL
Acetaminophen	17.3647	17.3165	17.4138
Acetaminophen caffeine butalbital	0.0141	0.0130	0.0154
Acetaminophen chlorpheniramine dextromethorphan pseudoephedrine	0.0101	0.0091	0.0111
Acetaminophen codeine	2.2798	2.2662	2.2927
Acetazolamide	0.1437	0.1403	0.1475
Acetylcysteine	0.2376	0.2328	0.2426
Acyclovir	0.6514	0.6431	0.6597
Adenosine	0.1002	0.0972	0.1037
Albumin	1.0432	1.0355	1.0514
Albuterol	6.4663	6.4473	6.4833
Albuterol ipratropium	0.0094	0.0084	0.0104
Alemtuzumab	0.0044	0.0038	0.0051
Alendronate	0.0044	0.0038	0.0051
Alfentanil	0.0252	0.0237	0.0268
Allopurinol	0.0827	0.0804	0.0848
Alprazolam	0.0476	0.0455	0.0496
Alprostadil	0.1436	0.1405	0.1470
Alteplase	0.3818	0.3765	0.3870
Aluminum	0.0068	0.0060	0.0077
Aluminum acetate	0.0145	0.0134	0.0157
Aluminum magnesium hydroxide	0.6240	0.6144	0.6343
Amantadine	0.0135	0.0124	0.0147
Amikacin	0.0988	0.0955	0.1019
Amiloride	0.0029	0.0024	0.0035
Aminocaproic acid	0.0738	0.0715	0.0760
Aminophylline	0.1536	0.1497	0.1574
Amiodarone	0.0517	0.0495	0.0539
Amitriptyline	0.0803	0.0776	0.0830
Amlodipine	0.1750	0.1712	0.1789
Ammonium chloride	0.0426	0.0405	0.0445
Amoxicillin	0.9451	0.9357	0.9549
Amoxicillin clavulanate	0.7150	0.7066	0.7230
Amphetamine complex	0.0221	0.0206	0.0237
Amphotericin b	0.1577	0.1539	0.1615
Ampicillin	8.9962	8.9632	9.0285
Ampicillin sulbactam	0.9867	0.9763	0.9967
Antithymocyte globulin	0.0114	0.0105	0.0125
Aprepitant	0.0222	0.0208	0.0237
Aprotinin	0.1555	0.1527	0.1583
Argatroban	0.0078	0.0069	0.0088
Arginine	0.0208	0.0194	0.0221
Aripiprazole	0.3525	0.3474	0.3575
Asparaginase	0.0340	0.0323	0.0360
Aspirin	0.4641	0.4583	0.4702
Aspirin codeine	0.0193	0.0164	0.0223
Atenolol	0.0740	0.0715	0.0765
Atomoxetine	0.1259	0.1223	0.1291
Atorvastatin	0.0105	0.0095	0.0116
Atovaquone	0.0135	0.0124	0.0146
Atracurium	0.2565	0.2520	0.2607
Atropine	1.2013	1.1923	1.2104
Azathioprine	0.0498	0.0478	0.0519

Azelastine	0.0088	0.0079	0.0098
Azithromycin	2.2722	2.2584	2.2849
Aztreonam	0.0370	0.0352	0.0389
Bacitracin	2.4278	2.4075	2.4478
Baclofen	0.1983	0.1940	0.2026
Balsalazide	0.0033	0.0027	0.0039
Barium sulfate	0.0807	0.0777	0.0836
Basiliximab	0.0052	0.0047	0.0058
Beclomethasone	0.0731	0.0705	0.0759
Belladonna opium	0.0159	0.0146	0.0171
Benzocaine	0.8149	0.8071	0.8228
Benzoin tincture	0.1183	0.1150	0.1219
Benzonatate	0.0230	0.0215	0.0245
Benztropine	0.1761	0.1712	0.1807
Beractant	0.4884	0.4818	0.4950
Betamethasone	0.1348	0.1316	0.1377
Bethanechol	0.0237	0.0223	0.0254
Bisacodyl	0.7536	0.7459	0.7628
Bleomycin	0.0285	0.0268	0.0301
Bosentan	0.0018	0.0013	0.0023
Botulinum toxin type a	0.0073	0.0065	0.0082
Bromocriptine	0.0067	0.0058	0.0075
Brompheniramine	0.0114	0.0104	0.0125
Budesonide	1.7875	1.7753	1.7991
Bumetanide	0.0631	0.0606	0.0654
Bupivacaine	2.1625	2.1496	2.1760
Bupivacaine epinephrine	1.3571	1.3465	1.3673
Bupropion	0.1636	0.1598	0.1672
Buspirone	0.0304	0.0287	0.0319
Busulfan	0.0096	0.0089	0.0104
Butorphanol	0.7702	0.7630	0.7776
Caffeine	0.9054	0.8984	0.9122
Caffeine sodium benzoate	0.0207	0.0195	0.0220
Calamine pramoxine	0.0671	0.0636	0.0708
Calcium acetate	0.0314	0.0296	0.0332
Calcium carbonate	0.2691	0.2631	0.2741
Calcium chloride	0.5885	0.5825	0.5941
Calcium gluconate	2.6932	2.6770	2.7094
Calfactant	0.2626	0.2579	0.2675
Captopril	0.1575	0.1541	0.1610
Carbamazepine	0.2077	0.2034	0.2124
Carbamide	0.0089	0.0079	0.0100
Carbamide peroxide otic	0.0223	0.0207	0.0240
Carbidopa levodopa	0.0048	0.0041	0.0055
Carboplatin	0.0393	0.0373	0.0413
Carboprost tromethamine	0.0467	0.0445	0.0488
Carvedilol	0.0160	0.0148	0.0174
Caspofungin	0.0372	0.0354	0.0391
Cefaclor	0.0500	0.0469	0.0533
Cefadroxil	0.0094	0.0086	0.0103
Cefamandole	0.0099	0.0089	0.0109
Cefazolin	4.2439	4.2298	4.2581
Cefdinir	0.2670	0.2620	0.2727
Cefepime	0.5625	0.5563	0.5697
Cefixime	0.0168	0.0155	0.0183

Cefotaxime	2.6960	2.6794	2.7125
Cefotetan	0.1673	0.1633	0.1715
Cefoxitin	0.6913	0.6848	0.6985
Cefpodoxime	0.0273	0.0257	0.0290
Cefprozil	0.0742	0.0714	0.0768
Ceftazidime	0.5675	0.5605	0.5744
Ceftizoxime	0.0269	0.0253	0.0285
Ceftriaxone	7.2806	7.2601	7.3014
Cefuroxime	0.8088	0.8004	0.8175
Celecoxib	0.0316	0.0300	0.0333
Cephalexin	0.3699	0.3641	0.3759
Cephradine	0.0199	0.0188	0.0210
Cetirizine	0.3999	0.3943	0.4061
Charcoal	0.0078	0.0070	0.0087
Chloral	0.7309	0.7232	0.7388
Chlorhexidine	0.4213	0.4157	0.4275
Chloroethane	0.0786	0.0759	0.0816
Chloroprocaine	0.0803	0.0746	0.0856
Chlorothiazide	0.2573	0.2529	0.2618
Chlorpromazine	0.0905	0.0875	0.0935
Cholestyramine	0.0175	0.0162	0.0188
Cholestyramine light	0.0995	0.0964	0.1028
Chromium	0.0027	0.0022	0.0033
Cidofovir	0.0030	0.0025	0.0036
Cimetidine	0.0637	0.0611	0.0664
Ciprofloxacin	0.3883	0.3824	0.3949
Ciprofloxacin dexameth otic	0.0468	0.0449	0.0490
Ciprofloxacin hc otic	0.0258	0.0243	0.0275
Cisapride	0.0028	0.0023	0.0034
Cisatracurium	0.3019	0.2962	0.3071
Cisplatin	0.0879	0.0853	0.0905
Citalopram	0.0831	0.0803	0.0860
Citrate phosphate dextr	0.0144	0.0132	0.0156
Cladribine	0.0048	0.0041	0.0055
Clarithromycin	0.1082	0.1049	0.1115
Clindamycin	2.4258	2.4135	2.4392
Clomipramine	0.0043	0.0037	0.0049
Clonazepam	0.2531	0.2486	0.2577
Clonidine	0.4157	0.4098	0.4216
Clopidogrel	0.0083	0.0074	0.0093
Clorazepate	0.0281	0.0265	0.0298
Clotrimazole	0.2030	0.1987	0.2076
Cocaine	0.0181	0.0167	0.0194
Codeine	0.1039	0.1005	0.1072
Colistimethate	0.0060	0.0052	0.0068
Collagenase	0.0096	0.0087	0.0106
Corticotropin	0.0178	0.0165	0.0190
Cosyntropin	0.0463	0.0442	0.0486
Cromolyn	0.0277	0.0259	0.0293
Cyclobenzaprine	0.0716	0.0692	0.0742
Cyclopentolate	0.1498	0.1462	0.1538
Cyclopentolate phenylephrine	0.6055	0.5991	0.6118
Cyclophosphamide	0.2462	0.2425	0.2500
Cyclosporine	0.0748	0.0724	0.0773
Cyproheptadine	0.0860	0.0831	0.0888

Cytarabine	0.1868	0.1828	0.1903
Daclizumab	0.0096	0.0088	0.0104
Dactinomycin	0.0367	0.0350	0.0383
Dalteparin	0.0141	0.0130	0.0152
Dantrolene	0.0138	0.0126	0.0150
Dapsone	0.0523	0.0501	0.0545
Darbepoetin albumin	0.0803	0.0777	0.0831
Darbepoetin alfa	0.0074	0.0065	0.0083
Daunorubicin	0.0450	0.0431	0.0469
Deferoxamine	0.0087	0.0078	0.0096
Desflurane	0.0143	0.0131	0.0155
Desloratadine	0.0119	0.0108	0.0130
Desmopressin	0.1451	0.1414	0.1488
Dexamethasone	3.0949	3.0809	3.1102
Dexamethasone neomycin	0.0345	0.0327	0.0362
Dexmedetomidine	0.1123	0.1093	0.1151
Dexmethylphenidate	0.0324	0.0307	0.0340
Dexrazoxane	0.0110	0.0100	0.0120
Dextran	0.0300	0.0283	0.0319
Dextroamphet	0.0401	0.0382	0.0418
Dextroamphetamin sulfate	0.0010	0.0007	0.0014
Dextroamphetamine amphetamine	0.1841	0.1803	0.1880
Dextromethorphan	0.0188	0.0176	0.0202
Dialysis Solution	0.0180	0.0170	0.0190
Diatrizoate	0.0705	0.0679	0.0731
Diatrizoate meglumine and diatrizoate sodium	0.2578	0.2533	0.2624
Diatrizoate sodium	0.0164	0.0150	0.0179
Diazepam	0.6160	0.6086	0.6231
Diazoxide	0.0085	0.0074	0.0096
Dibucaine	0.0791	0.0756	0.0823
Dicloxacillin	0.0048	0.0041	0.0056
Dicyclomine	0.0440	0.0419	0.0462
Digoxin	0.3024	0.2976	0.3075
Dihydroergotamine	0.0311	0.0297	0.0326
Diltiazem	0.0176	0.0164	0.0189
Dimenhydrinate	0.0123	0.0112	0.0134
Dinoprostone	0.2008	0.1964	0.2047
Diphenhydramine	3.5479	3.5310	3.5636
Diphenoxylate atropine	0.0336	0.0318	0.0353
Dipyridamole	0.0090	0.0080	0.0101
Divalproex sodium	0.4258	0.4204	0.4316
Dobutamine	0.2408	0.2365	0.2451
Docusate	2.0829	2.0714	2.0954
Docusate sodium casanthranol*	0.0519	0.0499	0.0543
Dolasetron	0.8004	0.7927	0.8084
Donnatal	0.0260	0.0245	0.0276
Dopamine	0.9528	0.9459	0.9602
Dornase	0.2793	0.2751	0.2833
Dorzolamide	0.0055	0.0048	0.0063
Dorzolamide timolol	0.0046	0.0040	0.0053
Doxapram	0.0082	0.0073	0.0090
Doxazosin	0.0058	0.0049	0.0067
Doxepin	0.0046	0.0039	0.0052
Doxorubicin	0.1437	0.1405	0.1470
Doxycycline	0.1583	0.1546	0.1620

Dronabinol	0.0294	0.0277	0.0310
Droperidol	0.0910	0.0879	0.0937
Drotrecogin alfa	0.0084	0.0075	0.0094
Duloxetine	0.0526	0.0503	0.0548
Edetate calcium disodium	0.0027	0.0022	0.0033
Edrophonium	0.1032	0.1003	0.1065
Edrophonium atropine	0.0179	0.0166	0.0192
Enalapril	0.2720	0.2674	0.2772
Enoxaparin	0.2993	0.2944	0.3044
Ephedrine	0.4288	0.4225	0.4347
Epinephrine	1.8896	1.8769	1.9019
Epoetin	0.3670	0.3616	0.3725
Epoprostenol	0.0049	0.0043	0.0056
Ertapenem	0.0800	0.0774	0.0827
Erythromycin sulfisoxazole	0.0222	0.0207	0.0236
Escitalopram oxalate	0.3452	0.3396	0.3506
Esmolol	0.0811	0.0783	0.0836
Esomeprazole	0.1757	0.1716	0.1795
Estradiol	0.0029	0.0024	0.0034
Estrogen conjugated	0.0209	0.0195	0.0224
Ethacrynic acid	0.0223	0.0208	0.0237
Ethambutol	0.0086	0.0077	0.0095
Ethosuximide	0.0065	0.0057	0.0074
Etomidate	0.1785	0.1746	0.1824
Etoposide	0.1964	0.1927	0.2000
Factor ix	0.0050	0.0044	0.0057
Factor viia	0.0399	0.0381	0.0419
Factor viii	0.0129	0.0118	0.0142
Famotidine	1.9043	1.8932	1.9164
Felbamate	0.0219	0.0205	0.0235
Fenoldopam	0.0049	0.0042	0.0056
Fentanyl	7.8643	7.8463	7.8825
Fentanyl bupivacaine	0.3988	0.3919	0.4054
Ferric subsulfate solution	0.0157	0.0143	0.0172
Ferrous sulfate	0.2376	0.2323	0.2423
Fexofenadine	0.0603	0.0579	0.0626
Filgrastim	0.2961	0.2912	0.3004
Flecainide	0.0082	0.0074	0.0091
Fluconazole	0.6871	0.6797	0.6943
Flucytosine	0.0023	0.0018	0.0028
Fludarabine	0.0076	0.0067	0.0084
Fludeoxyglucose	0.0035	0.0029	0.0041
Fludrocortisone	0.0388	0.0369	0.0407
Flumazenil	0.0795	0.0766	0.0822
Flunisolide	0.0713	0.0688	0.0738
Fluocinolone	0.0062	0.0055	0.0070
Fluocinonide	0.0105	0.0094	0.0114
Fluorescein	0.0117	0.0107	0.0129
Fluoride	0.0090	0.0081	0.0098
Fluorouracil	0.0119	0.0109	0.0130
Fluoxetine	0.4362	0.4308	0.4416
Fluticasone	1.1534	1.1438	1.1633
Fluticasone salmeterol	0.3252	0.3193	0.3304
Fluvoxamine	0.0176	0.0163	0.0189
Formaldehyde	0.0018	0.0013	0.0023

Formoterol fumarate	0.0141	0.0130	0.0153
Foscarnet	0.0022	0.0017	0.0027
Fosphenytoin	0.3859	0.3803	0.3912
Furosemide	2.2063	2.1954	2.2167
Gabapentin	0.1647	0.1607	0.1691
Gadodiamide	0.0139	0.0128	0.0151
Gadodiamide caldiamide	0.0113	0.0103	0.0122
Gadolinium	0.1770	0.1727	0.1808
Gadopentetate	0.3100	0.3045	0.3158
Gadoteridol	0.0034	0.0028	0.0040
Ganciclovir	0.0330	0.0315	0.0345
Gatifloxacin	0.0238	0.0223	0.0253
Gemtuzumab ozogamicin	0.0049	0.0042	0.0056
Gentamicin	6.6243	6.5943	6.6544
Gentian violet	0.0657	0.0603	0.0713
Glucagon	0.0620	0.0593	0.0645
Glutamic acid hcl	0.0094	0.0085	0.0103
Glutamine	0.0249	0.0232	0.0263
Glyburide	0.0097	0.0088	0.0107
Glycerin supplement	1.4441	1.4318	1.4563
Glycopyrrolate	2.3598	2.3481	2.3723
Granisetron	0.3124	0.3069	0.3184
Griseofulvin	0.0048	0.0041	0.0056
Guaifenesin	0.2439	0.2390	0.2489
Guaifenesin	0.0138	0.0126	0.0150
Guaifenesin codeine	0.0409	0.0391	0.0428
Guaifenesin dextromethorphan	0.1579	0.1540	0.1620
Guaifenesin pseudoephedrine	0.0156	0.0144	0.0168
Guanfacine	0.0517	0.0495	0.0541
Haloperidol	0.1698	0.1657	0.1739
Halothane	0.0019	0.0014	0.0024
Heparin	4.9935	4.9736	5.0118
Hespan	0.0574	0.0551	0.0596
Homatropine	0.0069	0.0061	0.0076
Human antithrombin iii	0.0034	0.0027	0.0040
Hyaluronate	0.0093	0.0084	0.0102
Hyaluronidase	0.0689	0.0663	0.0715
Hydralazine	0.1366	0.1331	0.1399
Hydrochlorothiazide	0.0445	0.0424	0.0465
Hydrocodone acetaminophen	1.9021	1.8901	1.9140
Hydrocodone chlorpheniramine	0.0185	0.0171	0.0197
Hydrocodone guaifenesin	0.0117	0.0107	0.0128
Hydrocortisone	0.8661	0.8571	0.8749
Hydrocortisone polyethylene glycol	0.0306	0.0290	0.0322
Hydrocortisone sodium phosphate	0.0126	0.0115	0.0137
Hydrocortisone sodium succinate	0.2438	0.2393	0.2486
Hydromorphone	0.9887	0.9798	0.9971
Hydroxychloroquine	0.0349	0.0332	0.0366
Hydroxyurea	0.0701	0.0676	0.0725
Hydroxyzine	0.5898	0.5830	0.5974
Hyoscyamine	0.0798	0.0772	0.0824
Ibuprofen	7.0231	7.0014	7.0477
Idarubicin	0.0020	0.0016	0.0024
Ifosfamide	0.0889	0.0861	0.0915
Imatinib mesylate	0.0036	0.0030	0.0042

Imipenem	0.0976	0.0946	0.1008
Imipenem cilastatin	0.0092	0.0083	0.0102
Imipramine	0.0277	0.0259	0.0295
Immune globulin cmv	0.0061	0.0053	0.0069
Immune globulin hepatitis b	0.9741	0.9524	0.9979
Immune globulin human ig	0.3512	0.3458	0.3568
Immune globulin rho d	0.1540	0.1506	0.1579
Immune globulin varicella zoster	0.0078	0.0069	0.0086
Indigotindisulfonate	0.0135	0.0124	0.0146
Indomethacin	0.3394	0.3347	0.3443
Infliximab	0.0224	0.0209	0.0238
Insulin	0.9112	0.9047	0.9177
Interferon gamma 1b	0.0013	0.0010	0.0017
Iodine	0.0118	0.0106	0.0130
Iodine 131	0.0020	0.0015	0.0024
Iodixanol	0.1221	0.1189	0.1253
Iohexol	0.1725	0.1685	0.1765
Iopamidol	0.0711	0.0686	0.0737
Iothalamate	0.0689	0.0664	0.0713
Iothalamate meglumine	0.0191	0.0176	0.0205
Ioversol	0.8322	0.8238	0.8417
Ioxaglate meglumine, ioxaglate sodium	0.0043	0.0036	0.0050
Ipratropium	2.5705	2.5572	2.5831
Ipratropium albuterol	0.3170	0.3115	0.3222
Irinotecan	0.0327	0.0312	0.0345
Iron	0.0799	0.0772	0.0826
Iron fumarate docusate sodium	0.0599	0.0574	0.0623
Iron gluconate	0.0322	0.0306	0.0339
Iron sucrose	0.2792	0.2678	0.2912
Iron sulfate	1.3472	1.3378	1.3572
Isoflurane	0.0739	0.0714	0.0767
Isoniazid	0.0190	0.0175	0.0205
Isoproterenol	0.0303	0.0287	0.0319
Isradipine	0.0212	0.0198	0.0227
Itraconazole	0.0158	0.0145	0.0170
Kanamycin	0.1172	0.1139	0.1206
Ketamine	0.6112	0.6041	0.6188
Ketoconazole	0.0266	0.0250	0.0282
Ketorolac	2.7080	2.6939	2.7214
L hyoscyamine sulfate	0.0106	0.0095	0.0116
L-cysteine	0.5114	0.5045	0.5179
Labetalol	0.2220	0.2173	0.2266
Lactobacillus	0.3785	0.3724	0.3845
Lactulose	0.1634	0.1596	0.1673
Lamivudine	0.0092	0.0082	0.0102
Lamotrigine	0.2658	0.2612	0.2710
Lanolin	0.3715	0.3626	0.3805
Lansoprazole	1.3459	1.3352	1.3567
Latanoprost	0.0055	0.0047	0.0062
Leucovorin	0.1572	0.1537	0.1609
Leuprolide	0.0031	0.0025	0.0036
Leuprolide depot	0.0130	0.0118	0.0141
Levalbuterol	2.3251	2.3116	2.3392
Levetiracetam	0.3640	0.3585	0.3694
Levocarnitine	0.3299	0.3246	0.3351

Levofloxacin	0.2998	0.2949	0.3049
Levothyroxine	0.2898	0.2847	0.2952
Lidocaine	10.9071	10.8456	10.9614
Lidocaine cardiac	1.0816	1.0672	1.0951
Lidocaine epinepherine tetracaine	0.0172	0.0160	0.0185
Lidocaine epinephrine	0.9888	0.9800	0.9975
Lidocaine prilocaine	3.4114	3.3841	3.4403
Lindane	0.0073	0.0066	0.0082
Linezolid	0.1021	0.0992	0.1052
Liothyronine	0.0097	0.0086	0.0106
Lisinopril	0.0698	0.0671	0.0723
Lithium	0.1299	0.1268	0.1334
Lomustine	0.0110	0.0100	0.0120
Loperamide	0.1130	0.1097	0.1162
Lopinavir ritonavir	0.0074	0.0067	0.0081
Loratadine	0.3524	0.3469	0.3578
Loratadine pseudoephedrine	0.0131	0.0121	0.0142
Lorazepam	2.1326	2.1197	2.1442
Losartan	0.0066	0.0057	0.0075
Lymphocyte immune globulin	0.0046	0.0039	0.0053
Mafenide	0.0088	0.0081	0.0097
Magnesia	0.0792	0.0763	0.0821
Magnesium	1.8079	1.7966	1.8191
Magnesium carbonate	0.0712	0.0686	0.0739
Magnesium hydroxide	0.5059	0.4995	0.5122
Mannitol	0.4196	0.4144	0.4247
Meclizine	0.0080	0.0072	0.0089
Medroxyprogesterone	0.1869	0.1828	0.1908
Megestrol	0.0434	0.0413	0.0455
Melatonin	0.0109	0.0099	0.0119
Melphalan	0.0064	0.0057	0.0070
Meperidine	1.5186	1.5078	1.5304
Meperidine promethazine	0.0179	0.0167	0.0193
Mepivacaine	0.0814	0.0784	0.0847
Mercaptopurine	0.1134	0.1098	0.1166
Meropenem	0.2533	0.2486	0.2580
Mesalamine	0.0783	0.0762	0.0806
Mesna	0.2663	0.2622	0.2702
Metaproterenol	0.0256	0.0238	0.0276
Metformin	0.0642	0.0617	0.0668
Methadone	0.2370	0.2325	0.2414
Methimazole	0.0039	0.0033	0.0045
Methocarbamol	0.0258	0.0243	0.0274
Methohexital	0.0058	0.0050	0.0065
Methotrexate	0.2510	0.2460	0.2553
Methyldopa	0.0118	0.0107	0.0130
Methylene blue	0.1219	0.1189	0.1248
Methylergon	0.1727	0.1681	0.1769
Methylphenidate	0.2887	0.2841	0.2940
Methylprednisolone	3.8684	3.8541	3.8824
Methyoxyflurane	0.0137	0.0125	0.0150
Metoclopramide	2.7527	2.7382	2.7673
Metolazone	0.0628	0.0605	0.0652
Metoprolol	0.0678	0.0653	0.0703
Metrizamide	0.0191	0.0176	0.0206

Metronidazole	0.9892	0.9804	0.9980
Mexiletine	0.0019	0.0014	0.0023
Micafungin	0.0069	0.0060	0.0077
Miconazole	0.0974	0.0939	0.1003
Midazolam	5.9647	5.9450	5.9831
Milrinone	0.3376	0.3339	0.3415
Minocycline	0.0215	0.0202	0.0230
Minoxidil	0.0092	0.0083	0.0102
Mirtazapine	0.0701	0.0676	0.0727
Misoprostol	0.1910	0.1867	0.1954
Mitomycin	0.0089	0.0081	0.0098
Mitoxantrone	0.0143	0.0132	0.0156
Mivacurium	0.3849	0.3788	0.3911
Modafinil	0.0275	0.0260	0.0292
Mometasone	0.2302	0.2253	0.2349
Montelukast	1.3879	1.3774	1.3988
Morphine	7.8720	7.8523	7.8894
Moxifloxacin	0.0864	0.0833	0.0898
Mupirocin	0.7781	0.7679	0.7886
Mycophenolate	0.0999	0.0970	0.1025
Nadolol	0.0053	0.0047	0.0061
Nafcillin	0.2814	0.2761	0.2863
Nalbuphine	0.5919	0.5843	0.6004
Nalmefene	0.0079	0.0069	0.0089
Naloxone	0.6364	0.6235	0.6481
Naproxen	0.1802	0.1761	0.1845
Neomycin	0.0541	0.0512	0.0573
Neomycin polymixin bacitracin	0.7785	0.7628	0.7949
Neomycin polymyxin	0.1584	0.1541	0.1629
Neomycin polymyxin hydrocortisone	0.0406	0.0386	0.0426
Neostigmine	1.8181	1.8076	1.8292
Nesiritide	0.0052	0.0045	0.0058
Nevirapine	0.0040	0.0030	0.0052
Nicardipine	0.0591	0.0569	0.0615
Nicotine	0.1037	0.1006	0.1070
Nifedipine	0.1743	0.1702	0.1781
Nitazoxanide	0.0163	0.0152	0.0175
Nitrofuran	0.0766	0.0738	0.0791
Nitrofurantoin	0.0276	0.0259	0.0292
Nitroglycerin	0.1198	0.1166	0.1234
Nitroprusside	0.1820	0.1785	0.1852
Nitrousoxide	0.0268	0.0251	0.0285
Norepinephrine	0.0778	0.0753	0.0803
Norethindrone	0.0011	0.0008	0.0014
Nortriptyline	0.0124	0.0115	0.0136
Nystatin	1.7684	1.7551	1.7821
Nystatin triamcinolone	0.0645	0.0621	0.0671
Octreotide	0.0312	0.0295	0.0329
Ofloxacin	0.1301	0.1268	0.1336
Olanzapine	0.1792	0.1753	0.1832
Olopatadine	0.0064	0.0056	0.0071
Omeprazole	0.2379	0.2332	0.2431
Ondansetron	5.9153	5.8978	5.9336
Opium	0.0176	0.0163	0.0191
Orphenadrine	0.0110	0.0100	0.0119

Oseltamivir	0.1006	0.0978	0.1035
Oxacillin	0.2028	0.1986	0.2071
Oxcarbazepine	0.4198	0.4138	0.4263
Oxybutynin	0.1872	0.1837	0.1908
Oxycodone	0.6656	0.6546	0.6768
Oxycodone asprin	1.4261	1.4139	1.4381
Oxymetazoline	0.3675	0.3619	0.3730
Oxymorphone	0.0190	0.0176	0.0204
Oxytocin	2.1101	2.1015	2.1189
Palivizumab	0.9043	0.8965	0.9126
Palonosetron hcl	0.0028	0.0022	0.0033
Pamidronate	0.0106	0.0096	0.0116
Pancrelipase	0.0719	0.0697	0.0742
Pancuronium	0.3023	0.2972	0.3075
Pantoprazole	0.6516	0.6444	0.6596
Papaverine	0.1990	0.1949	0.2030
Paroxetine	0.0713	0.0685	0.0741
Pegaspargase	0.0572	0.0553	0.0593
Pegfilgrastim	0.0267	0.0251	0.0282
Penicillin G	0.5604	0.5520	0.5685
Penicillin v	0.1479	0.1442	0.1517
Pentamidine	0.0733	0.0707	0.0758
Pentobarbital	0.2541	0.2492	0.2591
Peptamen	0.0415	0.0397	0.0433
Permethrin	0.0406	0.0386	0.0426
Perphenazine	0.0055	0.0048	0.0063
Phenazopyridine	0.0623	0.0598	0.0648
Phenobarbital	0.8253	0.8168	0.8332
Phenol	0.1257	0.1221	0.1287
Phentolamine	0.0193	0.0179	0.0208
Phenylephrine	0.9449	0.9358	0.9540
Phenylephrine chlorpheniramine hydrocodone	0.0192	0.0178	0.0206
Phenylpropanolamine brompheniramine	0.0204	0.0192	0.0218
Phenylpropanolamine chlorpheniramine dextromethorphan	0.0315	0.0299	0.0333
Phenytoin	0.2990	0.2940	0.3043
Physiosol	0.0730	0.0705	0.0753
Physostigmine	0.2827	0.2705	0.2949
Pimecrolimus	0.0217	0.0202	0.0231
Piperacillin	0.0221	0.0207	0.0236
Piperacillin tazobactam	0.7983	0.7900	0.8060
Polycarbophil	0.0080	0.0071	0.0089
Polyethylene glycol electrolyte	1.1256	1.1160	1.1358
Polymyxin	0.0746	0.0720	0.0772
Polymyxin bacitracin	0.5896	0.5740	0.6057
Polymyxin trimethoprim	0.0771	0.0736	0.0807
Poractant	0.3449	0.3390	0.3508
Potassium acetate	0.7057	0.6972	0.7139
Potassium chloride	6.0098	5.9871	6.0320
Potassium iodide	0.0066	0.0058	0.0074
Potassium phosphate	0.9427	0.9344	0.9508
Potassium phosphate sodium phosphate	0.0030	0.0024	0.0036
Povidone iodine	0.1776	0.1735	0.1818
Pramipexole	0.0126	0.0115	0.0136
Pramoxine	0.2356	0.2309	0.2403

Pravastatin	0.0029	0.0024	0.0035
Prazosin	0.0155	0.0143	0.0168
Prednisolone	2.6815	2.6673	2.6951
Prednisone	0.9471	0.9391	0.9554
Pregabalin	0.0128	0.0117	0.0138
Primidone	0.0062	0.0055	0.0070
Probenecid	0.0032	0.0026	0.0038
Procainamide	0.0057	0.0050	0.0066
Procarbazine	0.0019	0.0015	0.0023
Prochlorperazine	0.1510	0.1472	0.1548
Promazine	0.0018	0.0014	0.0022
Promethazine	2.8213	2.8067	2.8358
Promethazine codeine	0.0235	0.0219	0.0250
Proparacaine	0.1250	0.1219	0.1284
Propofol	5.2665	5.2502	5.2826
Propoxyphene	0.0121	0.0110	0.0132
Propoxyphene acetaminophen	0.3345	0.3292	0.3403
Propranolol	0.1330	0.1294	0.1365
Protamine	0.1108	0.1085	0.1131
Protamine sulfate	0.1222	0.1197	0.1246
Protriptyline	0.2748	0.2630	0.2870
Pseudoephedrine	0.1316	0.1283	0.1353
Pseudoephedrine brompheniramine	0.0597	0.0574	0.0623
Pseudoephedrine brompheniramine dextromethorphan	0.0152	0.0140	0.0165
Pseudoephedrine carbinoxamine	0.0179	0.0167	0.0192
Pseudoephedrine carbinoxamine dextromethorphan	0.0669	0.0645	0.0695
Psyllium	0.0441	0.0420	0.0462
Pyrazinamide	0.0082	0.0073	0.0091
Pyridostigmine	0.0146	0.0135	0.0158
Quetiapine	0.0339	0.0321	0.0357
Quetiapine fumarate	0.4544	0.4485	0.4603
Ranitidine	3.8649	3.8462	3.8827
Rasburicase	0.0129	0.0119	0.0140
Remifentanil	0.1321	0.1288	0.1355
Reteplase	0.0130	0.0120	0.0140
Ribavirin	0.0024	0.0020	0.0029
Rifampin	0.1295	0.1261	0.1328
Risperidone	0.4903	0.4844	0.4970
Ritonavir	0.0038	0.0033	0.0043
Rituximab	0.0188	0.0175	0.0201
Rocuronium	2.5251	2.5126	2.5374
Ropivacaine	0.4723	0.4661	0.4791
Rosiglitazone	0.0038	0.0032	0.0045
Salmeterol	0.0081	0.0073	0.0091
Salmeterol xinafoate fluticasone	0.0922	0.0892	0.0953
Sargramostim	0.0060	0.0052	0.0068
Scopolamine	0.0770	0.0741	0.0795
Selenium	0.1645	0.1600	0.1690
Senna	0.3761	0.3704	0.3824
Senna docusate	0.3596	0.3538	0.3657
Sertraline	0.3556	0.3497	0.3614
Sevelamer	0.0158	0.0147	0.0170

Sevoflurane	0.2334	0.2284	0.2383
Sildenafil	0.0332	0.0312	0.0350
Silver nitrate	0.1641	0.1570	0.1718
Silver sulfadiazine	0.2268	0.2227	0.2308
Simethicone	1.0598	1.0476	1.0718
Sincalide	0.0205	0.0192	0.0219
Sirolimus	0.0225	0.0210	0.0239
Sodium acetate	0.7036	0.6963	0.7114
Sodium benzoate	0.0012	0.0009	0.0015
Sodium bicarbonate	1.9117	1.8987	1.9247
Sodium citrate	0.4290	0.4232	0.4349
Sodium ferric gluconate complex	0.0195	0.0181	0.0208
Sodium lactate	0.0582	0.0555	0.0610
Sodium phenylbutyrate	0.0013	0.0010	0.0017
Sodium phosphate	0.4896	0.4830	0.4959
Sodium sulfacetamide	0.0030	0.0025	0.0036
Sodium thiosulfate	0.0115	0.0104	0.0125
Somatropin	0.0102	0.0092	0.0112
Sotalol	0.0111	0.0102	0.0121
Spironolactone	0.3215	0.3167	0.3265
Stavudine	0.0030	0.0025	0.0035
Succimer	0.0016	0.0013	0.0020
Succinylcholine chloride	1.4648	1.4533	1.4755
Sucralfate	0.2498	0.2450	0.2548
Sufentanil	0.1612	0.1575	0.1651
Sulfacetamide op	0.0472	0.0446	0.0498
Sulfasalazine	0.0098	0.0088	0.0108
Sumatriptan	0.0230	0.0216	0.0244
Tacrolimus	0.1235	0.1201	0.1267
Tamsulosin	0.0141	0.0130	0.0151
Technetium-99m unspecified	0.1105	0.1072	0.1139
Tegaserod	0.0182	0.0170	0.0196
Temazepam	0.0451	0.0430	0.0470
Temozolomide	0.0079	0.0070	0.0088
Terbutaline	0.2410	0.2363	0.2459
Testosterone	0.0012	0.0009	0.0016
Tetracaine	0.2000	0.1960	0.2040
Tetracycline	0.0152	0.0140	0.0164
Theophylline	0.1044	0.1013	0.1074
Thioguanine	0.0249	0.0233	0.0264
Thiopental	0.1565	0.1526	0.1606
Thiotepa	0.0027	0.0022	0.0032
Thrombin	0.6414	0.6353	0.6475
Tiagabine	0.0073	0.0065	0.0081
Ticarcillin	0.0680	0.0660	0.0710
Ticarcillin clavulanate	0.1683	0.1642	0.1724
Timolol	0.0020	0.0016	0.0025
Tizanidine	0.0259	0.0243	0.0275
Tobramycin	0.4897	0.4827	0.4973
Tobramycin dexamethasone opthalamic	0.0463	0.0440	0.0488
Tolterodine	0.0179	0.0166	0.0192
Topiramate	0.3117	0.3063	0.3169
Topotecan hcl	0.0070	0.0061	0.0078
Tramadol	0.0537	0.0515	0.0564
Tranexamic acid	0.0061	0.0053	0.0070

Trazodone	0.2732	0.2684	0.2780
Tretinoin	0.0023	0.0018	0.0028
Triamcinolone	0.2345	0.2298	0.2392
Trifluridine	0.0060	0.0050	0.0069
Trihexyphenidyl	0.0076	0.0067	0.0085
Trimethobenzamide	0.2117	0.2071	0.2161
Trimethoprim	0.0023	0.0019	0.0029
Trimethoprim sulfamethoxazole	1.2482	1.2382	1.2578
Tromethamine	0.0799	0.0769	0.0827
Tropicamide	0.1975	0.1930	0.2019
Undecylenic acid	0.0152	0.0135	0.0171
Urokinase	0.0074	0.0066	0.0083
Ursodiol	0.2372	0.2327	0.2416
Vacc dipth teta pertussis polio	0.0416	0.0367	0.0463
Vaccine: diptheria, tetanus	0.1912	0.1872	0.1951
Vaccine: diptheria, tetanus, pertussis	0.1624	0.1585	0.1661
Vaccine: diptheria, tetanus, pertussis, haemophilus b	0.0092	0.0082	0.0102
Vaccine: diptheria, tetanus, pertussis, hepatits b, polio	0.1706	0.1671	0.1744
Vaccine: haemophilus b	0.2667	0.2623	0.2710
Vaccine: haemophilus b, hepatitis b	0.1685	0.1599	0.1762
Vaccine: hepatitis a	0.0105	0.0089	0.0120
Vaccine: influenza	0.2736	0.2686	0.2788
Vaccine: measles, mumps, rubella	0.0521	0.0499	0.0543
Vaccine: meningococcal	0.0135	0.0124	0.0146
Vaccine: pneumococcal	0.4110	0.4056	0.4167
Vaccine: polio	0.1465	0.1431	0.1499
Vaccine: rabies	0.0027	0.0021	0.0033
Vaccine: rubella	0.1250	0.1215	0.1286
Vaccine: tetanus	0.0432	0.0412	0.0453
Vaccine: varicella	0.0097	0.0087	0.0106
Valacyclovir	0.0236	0.0221	0.0252
Valganciclovir	0.0380	0.0364	0.0396
Valproic acid	0.2926	0.2875	0.2978
Valsartan	0.0055	0.0048	0.0062
Vancomycin	2.5602	2.5477	2.5719
Vasopressin	0.0603	0.0581	0.0626
Vecuronium	1.1062	1.0966	1.1155
Venlafaxine	0.0831	0.0805	0.0858
Verapamil	0.0208	0.0194	0.0221
Vinblastine	0.0463	0.0442	0.0484
Vincristine	0.2871	0.2823	0.2916
Vitamin a	0.1410	0.1375	0.1445
Vitamin b1	0.0451	0.0431	0.0472
Vitamin B12	0.0405	0.0386	0.0427
Vitamin b2	0.0049	0.0041	0.0056
Vitamin b6	0.0559	0.0538	0.0581
Vitamin B9	0.3827	0.3773	0.3881
Vitamin c	0.1096	0.1063	0.1127
Vitamin D	0.2026	0.1983	0.2068
Vitamin e	0.1142	0.1109	0.1174
Voriconazole	0.0648	0.0625	0.0673
Warfarin	0.0816	0.0789	0.0841

Zalepon	0.0048	0.0042	0.0055
Zanamivir	0.0037	0.0030	0.0043
Zidovudine	0.0663	0.0615	0.0710
Zinc	0.3416	0.3358	0.3471
Ziprasidone	0.1597	0.1560	0.1634
Zolmitriptan	0.1223	0.1190	0.1257
Zolpidem	0.4169	0.4107	0.4231
